# Supplementary material for: Nature’s antivenom: Combinations of conserved rattlesnake serum metalloproteinase inhibitors block the lethal action of viper venoms
Source: Proc Natl Acad Sci U S A. 2026 Jul 29;123(32):e2612168123. doi: 10.1073/pnas.2612168123 (PMC13462563; doi:10.1073/pnas.2612168123)
Supplement: Supplementary file 1 [file pnas.2612168123.sapp.pdf]

PNAS

Supporting Information for

Nature's Antivenom: Combinations of conserved rattlesnake serum metalloproteinase inhibitors block the lethal action of viper venoms

Sean B. Carroll[1*†], Fiona P. Ukken[1†], Yetunde A. Ayinuola[1], Luis Escalona[2,3], Montamas Suntravat[2,4], Elda E. Sanchez[2,4]

Corresponding author Sean B Carroll
Email: carrolls@hhmi.org

This PDF file includes:

Supporting Text
Figures S1 to S4

Other supporting materials for this manuscript include the following:

Datasets S1

Supplementary Methods

Recombinant protein production

All constructs and recombinant proteins were prepared commercially (GenScript, Piscataway, NJ, USA). The sequences encoding *C. atrox* FETUA proteins were based on sequences obtained from the genome of a *C. atrox* specimen (s238). To facilitate their expression as recombinant proteins, the endogenous signal peptide sequence (1-19 amino acids) was replaced with a sequence optimized for protein expression (MGWSCIILFLVATATGVHS) in mammalian cell lines. The synthetic genes were cloned into the mammalian expression vector pcDNA3.4 (Invitrogen, Carlsbad, CA, USA).

The recombinant FETUA protein expression plasmids were transiently transfected into CHO-S cells and grown in serum-free media to facilitate the isolation of the tag-free proteins. After five days, the 40-100 ml culture supernatants were harvested and the proteins (isoelectric points approximately 5.6-5.8) were isolated by applying the supernatants to a HiTrap Q Fast Flow anion-exchange column (Cytiva) that was equilibrated with a low salt loading buffer (20mM Tris pH 8.0, 0% NaCl (w/v)) and eluting with a linear salt gradient (20mM Tris pH 8.0, 0-30% NaCl (w/v). The purity of the recombinant proteins was analyzed by size-exclusion chromatography on a TSKgelG3000SWXL HPLC column developed with 0.1M Na_2SO_4 in 0.118M phosphate buffer pH 7.0 and estimated to be 95-99%. The samples were dialyzed against either Tris-buffered saline (TBS) or phosphate-buffered saline (PBS) and stored at -80°C.

Protein assay

The protein concentration of sera was determined by Bradford assay (1). FETUA and CroFab FAb protein concentrations were determined by measuring A_{280} in a Nanodrop microspectrophotometer using extinction coefficients calculated from the primary amino acid sequence of the mature FETUA proteins and IgG.

SDS-PAGE

Protein samples were resolved by SDS-PAGE using 4-20% Criterion TGX™ Precast Midi Protein Gel for 40 minutes at 200 V (Criterion Cell; Bio-Rad). Samples were prepared in Laemmli buffer with or without 2-mercaptoethanol to achieve reducing or non-reducing conditions, respectively, and heated for 10 min at 95°C.

Collagenase activity

Azocoll substrate (Sigma-Aldrich) was suspended at 5 mg/ml in 20 mM Tris-HCl pH 8.0, 2mM $CaCl_2$. Working dilutions of venom were prepared from the venom stock solution in 20 mM Tris-HCl pH 8.0, 2mM $CaCl_2$ at 1 mg/ml. Ten micrograms of venom was incubated with increasing amounts of inhibitor candidates in 50 µl microliters and incubated at 37 °C for one hour. *D. acutus* venom was preincubated with 2.5mM AEBSF (final) to inhibit serine protease activity before incubation with inhibitor candidates. The substrate (950 µl) was added to the enzyme-inhibitor solution and incubated at 37 °C for 60 minutes with agitation. Following incubation, samples were centrifuged at 15,000 x g (or RPM) for 2 min, the supernatant was collected, and absorbance measured at 520 nm. All assays were performed in triplicate.

Fibrinogenolytic assay

One microgram of venom was preincubated with protease inhibitors (1 mM AEBSF and/or 10 mM EDTA, final concentrations) for 15 min at room temperature in PBS. Subsequently, the venom was incubated with FETUA proteins (2 µg of each protein) at 37 °C for 30 min, the 50 µg of human fibrinogen (50 µg; Enzyme research laboratories) was added in a final reaction volume of 20 µl, and the enzyme–inhibitor mixture incubated with shaking at 37 °C for 1 h. Samples were then loaded onto 8-16% gradient SDS–PAGE gels and analyzed by Coomassie Blue staining.

Nidogen digestion assay

One microgram of venom was preincubated with protease inhibitors (1 mM AEBSF, final concentration) for 15 min at room temperature. Subsequently, the venom was incubated with FETUA proteins (2 µg of each protein) at 37 °C for 30 min, then 50 µg of Matrigel basement membrane extract (Corning) was added in a final reaction volume of 50 µl Tris-buffered saline, 2mM $CaCl_2$, and the enzyme–inhibitor mixture was incubated with shaking at 37 °C for 1 h. Samples were then loaded onto 4-20% gradient SDS–PAGE gels, transferred onto PVDF membrane, and analyzed by Western blotting using an affinity purified rabbit anti-human nidogen 1 antibody diluted 1:6000 (proteintech, 13766-1-AP) and an Alexa Fluor 680 tagged goat anti-rabbit IgG.

Serine protease activity Assay

C. atrox venom serine protease activity was determined using N_α-Benzoyl-L-arginine 4-nitroanilide hydrochloride (BapNA) chromogenic substrate. To measure the effect of the FETA proteins on venom serine proteases activity, 50 µg of venom was incubated with either 0 µg or 50 µg of individual FETUA proteins in 50mM Tris-HCl, 150mM NaCl, 10 mM EDTA, pH 7.8, in a total volume of 180 µl at 37°C for 30mins, in the wells of a 96-well microtiter plate. The reaction was initiated by the addition of BapNA to a final

concentration of 0. 5mM. Hydrolysis of BapNA was monitored by measuring absorbance at 405 nm every 15 secs for 4 mins at 37°C. The rates of the catalyzed reactions were determined from the slopes of the linear plots of absorbance versus time, and serine protease activity was expressed as change in absorbance at 405 nm per minute.

Phospholipase A_2 activity assay

C. atrox venom PLA_2 activity assay was performed using a phospholipase A2 activity kit (Abcam). To evaluate the effect of the FETUA proteins on venom PLA_2 activity, 1600 ng of venom was incubated with either 0 ng or 1600 ng of individual FETUA proteins in PBS in a total volume of 40 µl at 37°C for 30mins. Following incubation,10 µl aliquots of each incubation mixture were added to corresponding wells of a second 96-well microtiter plate containing 5 µl assay buffer (25 mM Tris-HCl, 10 mM $CaCl_2$,100 mM KCl, 0.3 mM Triton X-100, pH 7.5) and 10 µl of 10 mM 5,5-dithio-bis-(-2-nitobenzoic acid) (DNTB). The reaction was initiated by adding 200 µl of substrate solution (1.66 mM diheptanoyl thio-PC dissolved in assay buffer). Activity of PLA_2 was monitored by measuring absorbance at 414 nm every 15 secs for 4 mins at 25°C. The rates of the catalyzed reactions were calculated from the slopes of the linear plots of absorbance versus time, and PLA_2 activity was expressed as change in absorbance at 414 nm per minute.

Phylogenetic analysis

Sequences used in our protein phylogenies were deduced from the hypothetical translation of assembled genomic regions from *D. russelli* or genomic regions from NCBI databases (see the full list of sequences Data S1). For phylogenetic analyses, we employed sequences from the first six exons because of the variable presence/absence of a deletion

in the seventh exon of FETUA-2 and FETUA-3. To construct phylogenies, we aligned coding sequences using MAFFT v7.505 (2) with the automatic algorithm selection option (--auto), and their translated protein sequences were aligned using the global pairwise alignment strategy with iterative refinement (--globalpair --maxiterate 1000). IQ-TREE2 (3) was used to generate maximum likelihood phylogenetic trees from coding sequences and protein alignments with automatic model selection. Branch support was evaluated using 1000 ultrafast bootstrap replicates and 1000 SH-aLRT replicates.

Supplemental References

1. M. M. Bradford, A Rapid and Sensitive Method for the Quantitation of Microgram Quantities of Protein Utilizing the Principle of Protein-Dye Binding. *Anal. Biochem.* **72**, 248–254 (1976).

2. K. Katoh, D. M. Standley, MAFFT multiple sequence alignment software version 7: Improvements in performance and usability. *Mol. Biol. Evol.* **30**, 772–780 (2013).

3. B. Q. Minh, *et al.*, IQ-TREE 2: New Models and Efficient Methods for Phylogenetic Inference in the Genomic Era. *Mol. Biol. Evol.* **37**, 1530–1534 (2020).

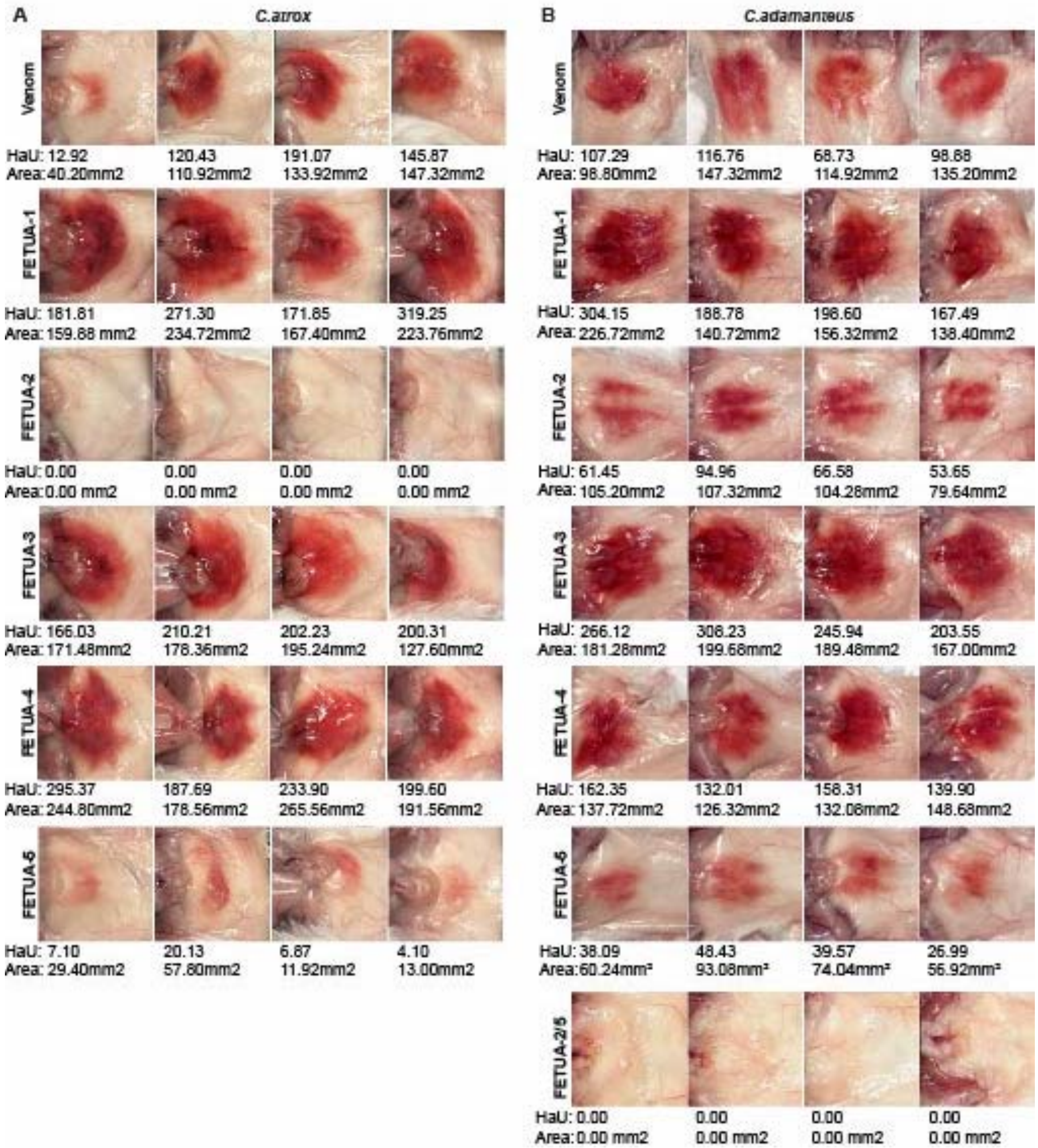

Fig. S1 Inhibition of rattlesnake venom hemorrhagic activity by selected FETUA proteins.

Groups of mice (n=4 per group) were injected subcutaneously with 1.5 µg of either (A) *C. atrox* venom or (B) *adamanteus* venom that was pre-incubated with either saline (venom control) or 10µg of individual or combinations of FETUA proteins indicated. The animals were sacrificed after 24 hr, the hemorrhage was photographed and hemorrhagic units (HaU) quantified by their area and intensity.

A**.** *C. atrox* venom hemorrhagic activity is completely inhibited by FETUA-2 and strongly inhibited by FETUA-5. FETUA-1, FETUA-3, and FETUA-4 enhance hemorrhage at the high protein concentration tested. The inhibition/enhancement calculations are tabulated in Table 1.

B. *C. adamanteus* venom hemorrhagic activity is partially inhibited by FETUA-2 and FETUA-5, and completely inhibited by the combination of FETUA-2 and FETUA-5. FETUA-1, FETUA-3, and FETUA-4 enhance hemorrhage at the high protein concentration tested. The inhibition/enhancement calculations are tabulated in Table 3.

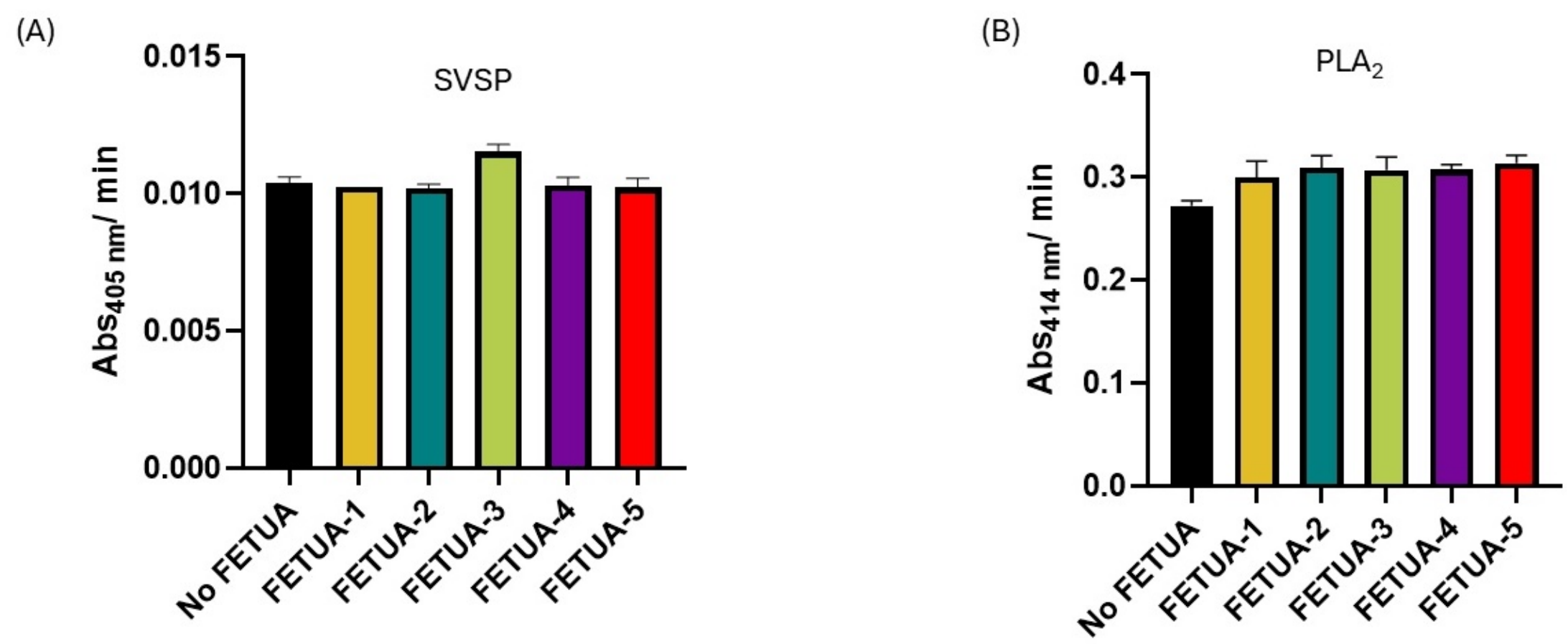

Fig. S2 FETUA proteins do not inhibit *C. atrox* venom serine protease (SVSP) and Phospholipase A2 activities.

(A) Bar plot showing the rate of hydrolysis of BapNA by *C. atrox* venom serine proteases in the presence of the indicated FETUA proteins.

(B) Bar plot showing the rate of hydrolysis of diheptanoyl thio-PC by of *C. atrox* venom PLA2 in the presence of the indicated FETUA proteins. Errors bars represent standard deviation of duplicate measurements from two separate assays.

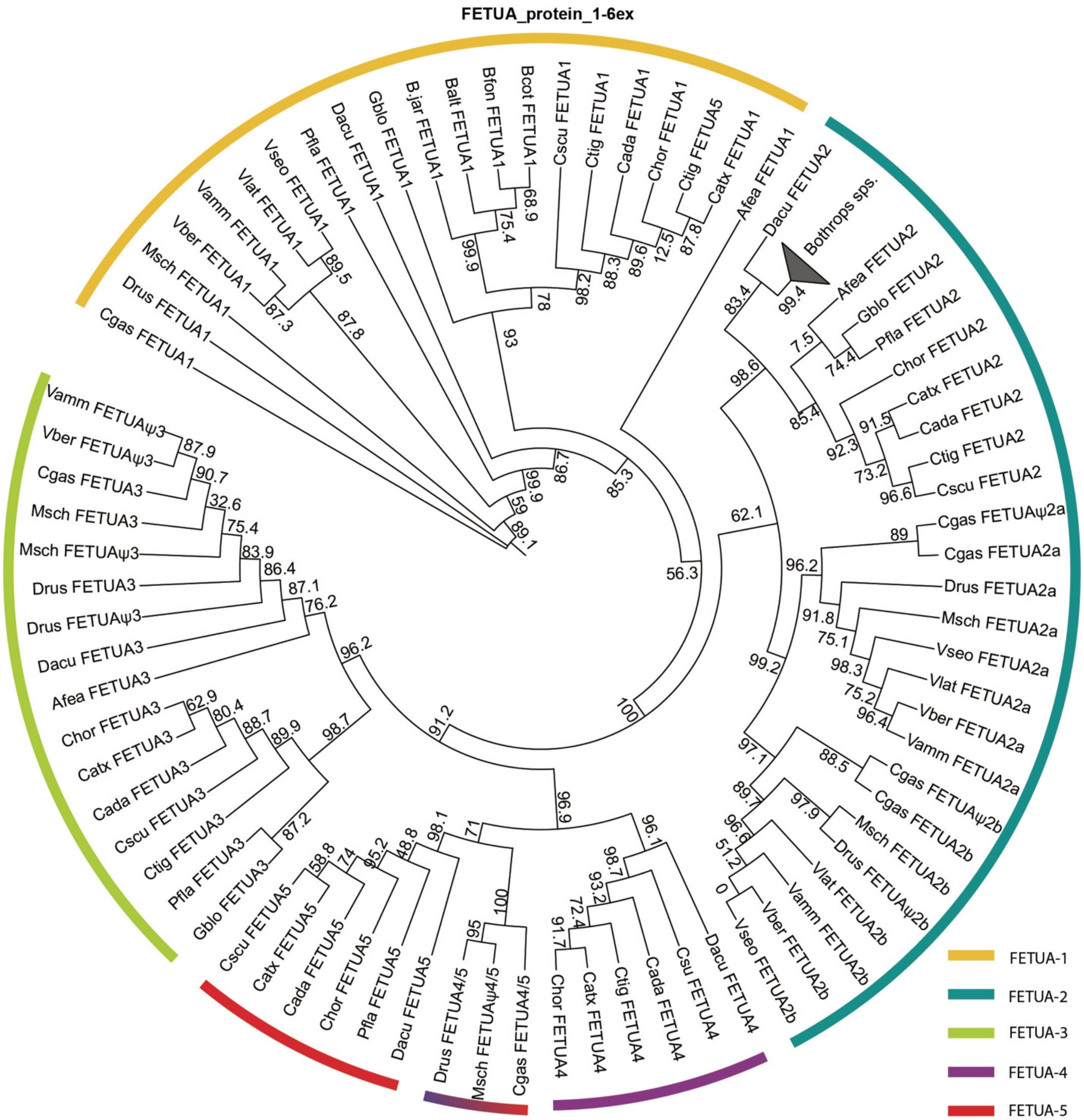

Fig. S3. All FETUA proteins belong to one of five paralog groups.
Phylogenetic analysis of FETUA protein sequences from vipers. The paralog groups are indicated by colors. Exon 7 sequences were excluded due to the variable presence/absence of deletions**.** The newly reported Viperinae FETUA proteins include up to two members of the FETUA-2 clade, FETUA-3, and a member of the FETUA-4/FETUA-5 clade that we refer to as FETUA-4/5. Sequences used are listed in Data S1.

```
Cgas_FETUA2b     MNSLVALLLLGQIIGSTLSSQVRGDIDCNDKDAKEWADMAVRYINEHKLHGYKLVLNVIK    60
Msch_FETUA2b     MNSLVALLLLGQIIGSTLSSQVRGDIDCNNKDAKEWADIAVRYINEHKLHGYKQVLNVIK    60
Vber_FETUA2b     MNSLVALLLLGQVIGSTLSSQVRGDIDCNDKDAKHWADIAVRYINEHKLHGYKQDLNVIK    60
Cgas_FETUA2a     MNFLVALLLLSQIIGSALSSQISGLVYCNDKEAKDWADKAVRYINEHKLHGYKQALNVIK    60
Vber_FETUA2a     MNSLVALLLLSQIIGSTLSSQVRGDIDCNDKDAKDWAGKAVRYINEHKLHGYKQDLNVIK    60
Msch_FETUA2a     MNSVVALLLLSQIIGSTLSSQVRGDIDCNDKDAKDWAGKAVRYINEHKLHGYKQALNVIK    60
Drus_FETUA2a     MNSLVALLLLSQIIGSTLSSQVRGDIDCNDKDAKDWAGIAVRYINEHKLHGYKQALNVIN    60
Dacu_FETUA2      MNSLVALLLLGQIIGSTLSSQVRGDLECDNKDAKEWADVAVRYINEHKLHGYKHALNVIK    60
Afea_FETUA2      MNSLVALVLLGQIIGSTLSSQVRGDLECDDKEAKEWAADAVRYINEHKLRGYKQALNVIK    60
Catx_FETUA2      MNSLVALVFLGQIIGSTLSSQVRGDLECDDKEAKEWADMAVRYINEHKLHGYKEALNVIK    60
                 ** :***::*.*:***:****: * : *::*:**.**  **********:***  ****:

Cgas_FETUA2b     NIHILTWSRDLVAGYLKLNLLETECHVLDPTPVKNCTVRPQHNHAVETDCDVKVIYNVMT    120
Msch_FETUA2b     NIRIVTWARDLVASYLKLNLLETECHVLDPTPVEKCTVRPQHNHTVETDCDVKVIYNVMT    120
Vber_FETUA2b     NIHILTWDRDLVAGYLKLNLLETECHVLDPTPVEKCTVRPQHNHTVEMDCDVKIIYNVMT    120
Cgas_FETUA2a     SIRILPWDGDLVAIYLKLNFVETECHVLDATPVENCIVRPRDNHTVETDCDVKIIYNIET    120
Vber_FETUA2a     NIHILPWDGDLVAIYLKLNFLETECHVLDPTPLENCTVRPQHNHAVKMDCDVKMIYDIET    120
Msch_FETUA2a     NIRILPWDGDLVAIYLKLNFLETECHVLDPTPVENCTVRPRDNHAVKIDCDVKIIYDIET    120
Drus_FETUA2a     NIRILPWNGDLVAIYLKLIFLETECHVLDPTPVKNCTVRPRDNHAVKIDCDVKIIYNIET    120
Dacu_FETUA2      NIVVVPWDGDLVAVFLKLNLLDTECHVSDSTPVENCTVREQHNHAVEMDCDVKIMFDIET    120
Afea_FETUA2      NILVVPWNGDLVAIFLKLNLLETECHVLDPTPVENCTVRPQHNHTVEMDCDAKIIFDIVT    120
Catx_FETUA2      NILVVPWNGDLVAVFLKLNLLETECHVLDPTPVENCTVRPQNNHAVEMDCDAKIMFDVVT    120
                 .* :: *  **** :*** ::***** * **:::* ** :.**:*: ***.*::::: *

Cgas_FETUA2b     LKDEVFVKCNSTPDSVENVQRNCPKCPILLPRNDPHVVDAVEYVLNKHNEKLSGHVYEVL    180
Msch_FETUA2b     LKDEVFVKCNSTPDSVENVQRNCPKCPILLPRNDPHVIDAVEYVLNKHNEKLSDHVYEVL    180
Vber_FETUA2b     LKDEVSVKCNSTTDSVENVQRNCPKCPILLPRNDPHVVDAVEYVLNKHNEKLSGHVYEVL    180
Cgas_FETUA2a     YKDEVFVKCISTPDSVENLQRICPKCPILLPRNDPHVVDAVEYVLNKHNEKLSGHVYEVL    180
Vber_FETUA2a     HKNDVFVKCNSTPDSVENVRQNCPKCPILLPLNDPHVVDAVEYVLNKHNEKLSGHVYEVL    180
Msch_FETUA2a     HKDNVFVKCNSTPDSVENVQQNCPKCPILLPRNDPHVVDAVEYVLNKHNEKLSGHVYEVL    180
Drus_FETUA2a     HKDNVFVKCNSTPDSVENVQRNCPKCPILLPRNDPHVVDAVEYVLNKHNEKLSGHVYEVL    180
Dacu_FETUA2      FKRDVFVKCHSTPDSVENVRRDCPKCLILLPPNDPRVVDSVEYVLSKHNEQLSGHIYEVL    180
Afea_FETUA2      FKQDVFVKCNSTPDSLENVRRNCPKCPILLPLNDPHVVDSAEYVLNKHNEKLSDHVYEVL    180
Catx_FETUA2      FKKDVFVKCHSTPDSVENVRRNCPKCPILLPRNDSHVVDSVEYVLNKHNEKLSGHIYEVL    180
                  * :* *** ** **:**::: **** **** ** :*:*:.****.****:**.*:****

Cgas_FETUA2b     EISRGQHKFEPEGYYVEFAIVQSNCTAQEAQDDHHHCHPNTAGEDHIGFCRATVFRSHAS    240
Msch_FETUA2b     EISRGQHTFEPEGFYVEFAIVQSNCTAQEAQDDHHHCHPNTAGEAHIGFCRATVFRSHAT    240
Vber_FETUA2b     EISRGQHTFEPEGFYVEFAIVQSNCTAQEAQDDHHHCHPNTAGEDHIGFCRATVFRSHAS    240
Cgas_FETUA2a     EISRGQHKFEPEGYYVEFAIVQSNCTAQEAQDDHHHCHPNTAGEDHIGFCRATVFRSHAS    240
Vber_FETUA2a     EISRGQHTFEPEGFYVEFAIVESNCTAQEAQDDHHHCHPNTAGEEHIGFCRATVIRSHAS    240
Msch_FETUA2a     EISRGQHKFEPEGFYVEFAIVQSNCTAQEAQDDHHHCHPNTAGEDHIGFCRATVFRSHAS    240
Drus_FETUA2a     EISRGQHKFEPEGIYVEFAIVQSNCTAQEAQDDHHHCHPNTAGEAYTGFCRATVFRSHAS    240
Dacu_FETUA2      EILRGQYKYEPEAFYVEFVIVEVNCTAQEAHDDHHHCHPNTAGEDHIAFCRATVFRSHVG    240
Afea_FETUA2      EISRGQHKYEPEAFYVEFAIVETNCTAQESHDDHHHCHPNTAGEDHIAFCRATVFRSHAS    240
Catx_FETUA2      EISRGQHKYEPEAFYVEFAIVEVNCTAQEAHDDHHHCHPNTAGEDHIGFCRATVFRSHAS    240
                 ** ***:.:***. ****.**: ******::************* : .******:***.

Cgas_FETUA2b     LEKPRHEQFESDCVIFDVKMGHAYSHLIKQHVGKYSTSPGDNTTVLNLAHSHNHTRDSQE    300
Msch_FETUA2b     LEKPKDEEFESDCVIFDVKKGHAHSHLIEQQIGKYSTSPGHNNTVLNLAHSHNDTRASHE    300
Vber_FETUA2b     LEKPKDEQFESDCVMFDVKEGHTHSYLIEQHVGKDSTSPGDNTTVLNLAHSHNHTRASHE    300
Cgas_FETUA2a     LEKPKDEQFESDCVIFDVKKGHAYSHLIEQHVGKYSTSSGDNTTVLNLAHSHNHTRDSQE    300
Vber_FETUA2a     LEKPKDEQFESDCVIFDVKKEHAHCYLIEQHVGKDSTSPGDNTNVLNLAHSHNHTRASHE    300
Msch_FETUA2a     LEKPKDEQFESDCVIFDVKKGHAHCYLIEHHVGKYSTSPGDNTTVLNLAHSHNHTRASHE    300
Drus_FETUA2a     LGKPKDEEYESDCEIFDVKKGHAHCYLIEHHVGKYSTSPGDNTTVLNLAHLHNHARASHE    300
Dacu_FETUA2      LEKPKDKKFESDCVILHVKDGHAHSHLIQQHIEKNSISPEHNITILNFIHPQNHTSTSH-    299
Afea_FETUA2      LEKPKDEKFESDCVIFDVKEGHAHSHLIEQHES--------------------------    273
Catx_FETUA2      LEKPKDEKFESDCVILHVKEGHAHSHLIEHHTGKYSTSPGSN-----------------    282
                 * **:.:::**** ::.**  *::.:**:::

Cgas_FETUA2b     SHSDECMVGCPVAFVKKEVPTDISDHHTPPVKGCPGRVLHFHL*        343
Msch_FETUA2b     SHSDECVVECPVTFVKKEVPTDISDHHTPPVKGCPGRVHHFKL*        343
Vber_FETUA2b     FHSDECVVECPVAFVKKEVPTDISDHHTPPAKGCPGRVLHFKL*        343
Cgas_FETUA2a     SHSDECVVGCPVAFVKKEVPTDISDHHTPPVKDCPGRVLHLNP*        343
Vber_FETUA2a     SHSDECVVGCPVAFVKKEVPTDISDHDTPSVKGCPGRVLHFNL*        343
Msch_FETUA2a     SHSDECVVGCPVAFVKKEVPTDISDHDTPPVKGCPGRVLHFNL*        343
Drus_FETUA2a     SHSEECVVGCPVAFVKKEVPTDISDHHTPPVKGCPGRVLHFNL*        343
Dacu_FETUA2      -ESHEHAAEVPVDFVKKELPTDISNRHTPPVKGCPGKVHHFEL*        341
Afea_FETUA2      -HSDECVAEALFAFVKKERPTDISDRHTHPVKGCPGKVHHFEL*        315
Catx_FETUA2      -STDECVVQCPVAFVNKELPTDISDHHTTPVKGCPGKVFHFQL*        324
                   :.*  .   . **:** *****::.*  .*.***:* *:. *
```

A

B

```
C.atx_FETUA3      MNSLVALVLLGQIIGSTLSFQLAGNMDCNTKGTKDWADIGVRYINEHKLHGYKQALNIIK  60
A.fea_FETUA3      MNSLVALVLLGHIIGSTLSFQLPGDMDCNTKGTKDWADIGVRYINEHKLHGYKQALNVIK  60
D.rus_FETUA3      MNSLVVLLLLGQIIGSTLSFQLRGDMDCNSKGTKDWANIGVSYINDHKLHGYKQALNIIK  60
C.gas_FETUA3      MNALVALLLLGQIIGSTLSFQLVGNMDCNTKGTKDWADIGVRYINDHKLHGYKQALNRIK  60
M.sch_FETUA3      MNSLVALLLLGQIIGSTLSFQLAGDMDCNTKGTKDWANIGVRYINDHKLHGYKQALNIIK  60
                  **:**.*:***:********** *:****:*******:*** ***:*********** **

C.atx_FETUA3      IFRLLPSDGRSVIFHFKLNLLETECHVLDPTPLENCTVRQQNNHAVEMDCNVRIIHDIAT  120
A.fea_FETUA3      IFRLLPSDGRSVIFYFKLNLLETECHVLDPTPVEKCTVRPQHNHAVEMDCNVKIIYDIVT  120
D.rus_FETUA3      FFDLLPSGGISETFYLKLNLLETECHVLDPTPVEKCTVRPQHNHTVEMDCNVIIIYNIET  120
C.gas_FETUA3      IFRLLPSGGIVETFYFKLNFLETECHVLDPTPVEKCTVRPQHNHTVEMDCDVIIIYNIDT  120
M.sch_FETUA3      IFRLLPSDGISETFYFKLNLLETECHVLDPTPVEKCTVRPQHNHTVEMDCNVIIIYNIET  120
                  :* ****.*    *::***:************:*:**** *:**:*****:* **::* *

C.atx_FETUA3      FEDEVFVKCNSTPDSVENVRRNCPKCPILLPPNDPHVVDSVEYVLNKHNEKLSGHIYEVL  180
A.fea_FETUA3      FKDEVFVKCNSTPDSLENVRRNCPKCPILLPLSDPHVVDSAEYVLNKHNEKLSDHVYEVL  180
D.rus_FETUA3      FRKTVFVKCNSTTDSVENVQRNCPKCPILLSRNDPDVIDAVEYVLNKNNEKLSGHVYEVL  180
C.gas_FETUA3      FGKEVFAKCNSTADSVENVQRNCPKCPILLPRNDPHVVDAVEYVLNKHNEKLSGHVYEVL  180
M.sch_FETUA3      FREKVFVKCNSTTDSVESVQRNCPKCPILLPRNDPHVVDAVEYVLNKHNEKLSGHVYEVL  180
                  * . **.***** **:*.*:**********  .**.*:*:.******:*****.*:****

C.atx_FETUA3      EISRGQHKYEPEAFYVEFAIVEVNCTAEEAHDDHHDCHPNTDGENHIGFCRSTVFRSHAS  240
A.fea_FETUA3      EISRGQHKYEPEAFYVEFAIVETNCTAQESQDDHHHCHPNTAGEDHIGFCRATVFRSHAS  240
D.rus_FETUA3      EISRGQHTFEPEGIYVEFAIVQSNCTAQEAQDDHHHCYPNTAGEDHIGFCRATVFRSHAS  240
C.gas_FETUA3      EISRGQQKFEPEGYYVEFAIVQSNCTAQEAQDDHHHCHPNTAGEDHIGFCRATVFRSHAS  240
M.sch_FETUA3      EISRGQHKFEPEGFYVEFAIVQSNCTAQEAQDDHHHCHPNTAGEDHIGFCRATVFRSHAS  240
                  ******:.:***. *******: ****:*::****.*:*** **:******:********

C.atx_FETUA3      LEKPKDEKFESDCVILHVKKGHAYSHLIEHHVGKYNTSPGRNS-----------------  283
A.fea_FETUA3      LEKPKDEKVESDCVIFDVKEGHAHSYLIEQHVGKYSVSSGHNSTVLNLAHSHNHTRPSHE  300
D.rus_FETUA3      LGKPKDEEYEADCEIFDVKKGHAYSHLIKHHVAKYSTSPGDNTTVLNLAHSHNHARASQE  300
C.gas_FETUA3      LEKPKDEQFESDCVIFDVKMGHAYSHLIEQHVGKYSTSPGDNTTVLNLAHSHNHTSDSQE  300
M.sch_FETUA3      LEKPKDEQFESDCVIFDVKNGHAYSHLIKHHIGKYSTSPGDNTTVLNLAHSHNHTRASQE  300
                  * *****: *:** *:.** ***:*:**::*:.**..* * *:

C.atx_FETUA3      --TDECVVQCPVAFVNKEVPKDISDHDTPP-VEGCPGKALHFQL* 324
A.fea_FETUA3      SHSDECVMECPVAFVKKEVPTDISDHDTPP-VEGCPGRVLHFKL* 343
D.rus_FETUA3      SHSEECVVECPVAFVKKEVPTYISDHDIPPVVEGCPGRALHFNL* 344
C.gas_FETUA3      SHSDECMVGCPVAFVKKEVPTDISDHDTPP-VKGCPGRVLHFHL* 343
M.sch_FETUA3      SHSDECVVGCPVAFVKKEVPTYLSDHDTPP-VKGCPGRVLHFKL* 343
                    ::**:: ******:****. :**** ** *:****:.***:**
```

```
D.rus_FETUA4/5      MNSLVALLLLSQIIGSTLNYIVTKKHLNCNSSGATGKANRAVHYINEHNRHGYKQALNEI 60
C.gas_FETUA4/5      MNSLVALLLLSQIIGSTLSYIVTKQHLNCNSSGAKGKADRAVHYINEHNLHGYKQALNEI 60
C.atx_FETUA5        MNSLVALVLLGQIIGSTHSYMVREEDLNCNSKAAKYRADQAVHYINEHNLHGYKQALNII 60
D.acu_FETUA5        MNSLVVLVLLGQVIGSTHSSMVKEEDLNCNSKAAKYRADQAVDYINEHNLHGYKQALNVI 60
C.atx_FETUA4        MNSLVVLVLLGQIIGSTHSYMMREEDMNCNSKAAKYRADQAVRYINEHKLHGYKQALNII 60
D.acu_FETUA4        MNSLVALLLLGQIIGSTLSYLVKEEDLNCNSKAAKYRADQVVRYINDHKLHGYKQALNVI 60
                    *****.*:**.*:**** . :: ::.:****..*. :*::.* ***:*: ******** *

D.rus_FETUA4/5      EIIRLIPSGGRIVTFYLKLNLLETVCHVLDPTPVEKCTVRPQHYHAVESDCDVKVVFNYV 120
C.gas_FETUA4/5      EIIRLIPSGGRTVIFYLKLNLLETECHALDPTPVEKCTVRSQHNHAVESNCDVKVIYNYV 120
C.atx_FETUA5        KTIRLLPSDGLLVVIYLKLNLLETECHVLDPTPVENCTVRQQNNHAVETDCDVKIIYNIE 120
D.acu_FETUA5        KIIRLLPSDGLSVIFNLKLNLLETKCHVLDPTPVENCTVRPQLNHAVETDCDVKIIYNIA 120
C.atx_FETUA4        KTFHLLPPERFTTVFYLKLNLLETECHVLDPTPVENCTVRPQNNHAVEMDCDVKINFNFE 120
D.acu_FETUA4        KIFHLLSPERFSTVFYLKLNLLDTECHVLDQTPVEKCTVRPQHNHAVEMDCDAKIIFNYD 120
                    : ::*:      . : ******:* **.** ****:**** *  **** :**.*: :*

D.rus_FETUA4/5      TFEEEFFVKCNSIPDSVENVQRNCPKCPILLPLNDPHVVDAVEYVLNKHNEKLSGHVYEV 180
C.gas_FETUA4/5      TYQEQFFVKCNSIPDSVENVQRNCSKCPILLPLNDPHVVDAVEYVLNKHNEKLSGHVYEV 180
C.atx_FETUA5        TFKDEFLVKCHSTPGSVEKILQDCPKCPILLSLSDPHVVDSVEYVLNKHNEQLSGHVYEV 180
D.acu_FETUA5        TFKDEFLVKCHSTPGSVENILRDCPKCPLLLPLSDPHVVDSVEYVLSKHNEKLSGHIYEV 180
C.atx_FETUA4        FFKTEIFVKCNSTPDSVENVQRNCPKCPILLPPNDPHVINSVEYMLNKHNEKLSGHVYEV 180
D.acu_FETUA4        IYKDYIFVKCNSTPDSVENVRRNCPKCPILLPPNDPRVVDSVEYVLSKHNEQLSGHIYEV 180
                     ::  ::***:* *.***:: ::* ***:**  .**:*:::***:*.****:****:***

D.rus_FETUA4/5      LEISRGQHKFEPEGIYVEFAIVQSNCTAQEAQDDHHHCHPNTARKDHIGFCRATVFRSNA 240
C.gas_FETUA4/5      LEISRGQQKFEPEGYYVEFAIVQSNCTAQEAQDDHHHCHPNTAGEDHIGFCRATVFRSHA 240
C.atx_FETUA5        LEISRGQHKYEPEAFYVEFAIVEVNCTAEEAHDDHHHCHPNTDGENHIGFCRATVFRSHA 240
D.acu_FETUA5        LEISRGQHQYEPEAFYVEFVIVEVNCTAQEAHDDHHHCHPNTAGEDHIAFCRATVFRSHA 240
C.atx_FETUA4        LEISRGQHKYEPEAFYVEFAIVEVNCTAEEAHDDHHHCRPNTAGEDHIGFCRATVFRSHA 240
D.acu_FETUA4        LEISRGQYKYEPEAFYVEFVIVEVNCTAQEAHDDHHHCHPNTAGEDHIAFCRATVFRSHA 240
                    ******* ::***. ****.**: ****:**:******:***  ::**.*********:*

D.rus_FETUA4/5      SLEKPKDEEYESDCVIFDVKKGRAHSHLIDHHIGKYSTSPGDNSTVLNLAHSHNDTRGSP 300
C.gas_FETUA4/5      NLEKPKDEQFESDCVILGVKYGHAHAHLIDHHIGKYSTSPGDNSTVLNLVHSHNDTRASP 300
C.atx_FETUA5        SLEKPKDEMSESDCVILDVKEGHAHSHLIEHHTGKYSISPGHNNTVLNLAHSHNHTSASH 300
D.acu_FETUA5        SLEKPKDEKFESDCVILHVKGGHAHSHLIQHHTGKYSTSPGHNSTILNVAHSHNYTSASH 300
C.atx_FETUA4        SLEKPKDEMFESDCVILHVKKGHAHSHLIQQHIGKYNISPGHHNTVLNLAHSHNDTRASQ 300
D.acu_FETUA4        SLEKPKDEKFESDCVILNVKRGHAYSQLIQHHVEKNSISPGHNSTVLNLAHSHNHTRASH 300
                    .*******  ******: ** *:*:::**::*  * . ***.:.*:**:.**** * .*

D.rus_FETUA4/5      QSHS------------------------EQPVDGCPGEVHHFKL*        320
C.gas_FETUA4/5      EFHSSE----------------------KTPVDGCPGKVRHFKL*        322
C.atx_FETUA5        ESHSHEHVTEVPVAVAKREVPTDIPHHHTPSVDGCPGKVLHFKL*        344
D.acu_FETUA5        ESHSHEHVAEVPVDSVKKEVPTDIPHHHTPPVDGCPGKVLHFKL*        344
C.atx_FETUA4        ESHSDECVVECPVAFVKKEVPTHIPHH-THPVEGCPGKVHHFKL*        343
D.acu_FETUA4        ESHSDECVVECPVAFVKKNIQTNIPHH-THPVEGCPGRVFHFQL*        343
                    : **                          *:****.* **:**
```

C

Fig. S4. Alignments of viper FETUA proteins reveal strong sequence conservation throughout each protein clade.

A. Selected proteins from the FETUA-2 clade.

   The viper FETUA-2 proteins exhibit 74-77% identity to *C. atrox* FETUA-2.

B. Selected proteins from the FETUA-3 clade.

   The viper FETUA-3 proteins exhibit 73-78% identity to *C. atrox* FETUA-3.

C. Selected proteins from the FETUA-4 and FETUA-5 clade.

   The viper FETUA-4/5 proteins exhibit 68-70% identity to *C. atrox* FETUA-5.

Species abbreviations are M. sch, *Macrovipera schweizeri*; C. gas, *Cerastes gasperttii*; V. ber. *Vipera berus*; D. Rus, *Daboia russelli*; D. acu, *Deinagkistrodon acutus*; A. fea, *Azemiops feae;* C. atx, *Crotalus atrox*.

Symbols: Conserved amino acids are indicated by asterisks (*), sites with conservative substitutions are denoted by a colon (:), and sites with semi-conservative substitutions are denoted with a period (.)

Dataset S1 FETUA protein sequences used in this study.

>D.acu_FETUA1
MNSLVALLLLGQIIGCTFSHHLTSQVDCNGEDAEKWGDMAVHYINEHNLHGYK
QALNVIKEIHVLPRRPHGEIVFIEIEVLETQCHVLDETPVENCTVRPQHYHAVGGD
CDVKIIHHEGVDKVVGAKCHSDPDSVEDVRRNCPNCPILLPLSDPRVVDCVEYV
LSKHNEKLSGHIYEVLEISRGQHQYEPEAFYVEFVIVEVNCTAQEAHDDHHHCHP
NTAGEDHIAFCRATVFRSLASLEKPKDEKFESDCVILNVKEGHAHSHLIQHHVGK
YSISPGHNNTVLNLAHSHNHTSASHESHSHEHVAEVPVAVAKREVPTDIPHHHM
HSHPVKLCPGKVHHFKL*

>D.acu_FETUA2
MNSLVALLLLGQIIGSTLSSQVRGDLECDNKDAKEWADVAVRYINEHKLHGYK
HALNVIKNIVVVPWDGDLVAVFLKLNLLDTECHVSDSTPVENCTVREQHNHAVE
MDCDVKIMFDIETFKRDVFVKCHSTPDSVENVRRDCPKCLILLPPNDPRVVDSVE
YVLSKHNEQLSGHIYEVLEILRGQYKYEPEAFYVEFVIVEVNCTAQEAHDDHHH
CHPNTAGEDHIAFCRATVFRSHVGLEKPKDKKFESDCVILHVKDGHAHSHLIQQ
HIEKNSISPEHNITILNFIHPQNHTSTSHESHEHAAEVPVDFVKKELPTDISNRHTPP
VKGCPGKVHHFEL*

>D.acu_FETUA3 (partial)
MNSLVALLLLGRIIGSTLSFQLAGDMDCMTKGTKDWADNVVRYINDNKLHGYK
QALNVIKIFHLPPSDALSVKFYFELNFLDTECHVLDQTPVENCTVRPQNNHAVEM
DCDAKIIFNYDIYKDYIFVKCNSTP

>D.acu_FETUA4
MNSLVALLLLGQIIGSTLSYLVKEEDLNCNSKAAKYRADQVVRYINDHKLHGYK
QALNVIKIFHLLSPERFSTVFYLKLNLLDTECHVLDQTPVEKCTVRPQHNHAVEM
DCDAKIIFNYDIYKDYIFVKCNSTPDSVENVRRNCPKCPILLPPNDPRVVDSVEYV
LSKHNEQLSGHIYEVLEISRGQYKYEPEAFYVEFVIVEVNCTAQEAHDDHHHCHP
NTAGEDHIAFCRATVFRSHASLEKPKDEKFESDCVILNVKRGHAYSQLIQHHVEK
NSISPGHNSTVLNLAHSHNHTRASHESHSDECVVECPVAFVKKNIQTNIPHHTHP
VEGCPGRVFHFQL*

>D.acu_FETUA5
MNSLVVLVLLGQVIGSTHSSMVKEEDLNCNSKAAKYRADQAVDYINEHNLHGY
KQALNVIKIIRLLPSDGLSVIFNLKLNLLETKCHVLDPTPVENCTVRPQLNHAVET
DCDVKIIYNIATFKDEFLVKCHSTPGSVENILRDCPKCPLLLPLSDPHVVDSVEYV
LSKHNEKLSGHIYEVLEISRGQHQYEPEAFYVEFVIVEVNCTAQEAHDDHHHCHP
NTAGEDHIAFCRATVFRSHASLEKPKDEKFESDCVILHVKGGHAHSHLIQHHTGK
YSTSPGHNSTILNVAHSHNYTSASHESHSHEHVAEVPVDSVKKEVPTDIPHHHTP
PVDGCPGKVLHFKL*

>P.fla_FETUA1

MNSLVALLLLGQIIGCTFSHHLPSHVPVDCNGEDAEKWADLAVDYINEHNLHGY
KYAVNVINEIFVLPRRPHGKMILLELNLLETECHVLDQTPIKNCTVRPPHYHAVE
GDCDVKIIHDEDVDKVVAAKCYSNPDSVEDVQQNCPKCPILLSLTDPHVVDSVE
YVLEKHNAKLSGHIYEVLEISRGQHKYEPEAYYLEFVIVEVNCTAQEAHDDHHQ
CHPYTAGEEHIGFCRATVFRSHASLEKPKDEKFESDCVIFDIKEGHAHSHLIEHHV
GKYSTSPGYNNTVLNLVHSHNHTSASHESHSHEHVAEVPVAVAKREVPTDIPHD
HTHPVKLCPGKVHHFKL*

>P.fla_FETUA2/HSF
MNSLVALVLLGQIIGSTLSSQVRGDLECDDKEAKNWADDAVRYINEHKLHGHK
QALNVIKNICVVPWNGDLVAVFLELNLLETECHVLDPTPVEKCTVRQQHNHAVE
MDCDAKIMFNVETFKRDVFVKCHSTPDSVENVRRNCSKCPILLPPNNPHVVDSV
EYVLNKHNEKLSGHIYEVLEISRGQHKYEPEAYYLEFVIVEINCTAQEAHDDHHQ
CHPYTAGEDHIAFCRSTVFRSHASLEKPKDEKFESDCVILDVKDGHAHSHLIQQHI
EKNSISPEHNITILNFVHPDNHTSTSHESHEHVAEVPVVFVKKELPTDISDHHTTPV
KGCPGKVHHFEL*

>P.fla_FETUA3/HLP
MNSLVALVLLGQIIGSTVSFQLGPNMDCNTKGTKDWADIGVHYINEHKLHGYKQ
ALNVIKIFRLLPSDGRSVIFHFNLNLLETECHVLDSTPVENCTVRPQHNHAVEMD
CNVRIIHDITTFEDEVFVKCSSTPGSVENILRDCPKCPILLSPNDPHVVDSVEYVLN
KHNEKLSGHIYEVLEISRGQHKYEPEAYYLEFVIVEINCTAQEAHDDYHQCHPYT
AGEDHIAFCRSTVFRSHASLEKPKDEKFESDCVILDVKEGHSHSHLIEHHVGKYS
TSPGYNSTDECVVECPVAFVNKEVPTDISDHNTPPVKGCPGRVLHFQL*

>P.fla_FETUA5
MNSLVALVLLGQIIGSTHSYMVKEEDLNCNSKGAKY*ADQAVRYINEHNLHGY
KQALNVIKIFRLLPSAGFSVIIYLKLNLLETECHVLDSTPVENCTVRQQHNHAVET
DCDVKIIYNIMTFKDEFLVKCHSTPGSVENILRDCPKCPILLSLSDPHVVDSVEYV
LEKHNAKLPGHIYEVFEISRGQHKYEPEALYLEFVIVEVNCTAQEAHDDHHQCHP
YTAGEDHIAFCRSTVFRSHASLEKPKDEKFESDCVILDVKEGHAHSHLIQQHIEKN
SISPEHNITILNFVHPHNHTSTSHESHEHVADVPVAFVKKELPTDISDHHTTPVKG
CPGKVHHFEL*

>G.blo_MSF
MHFLVALVLLGQIIGSTLSSQVRGDLECNDREAKEWADQAVRYINEHKLHEYKQ
ALNVIKNIVVVPWNGDLVAVFLKLNLLETECHVLDPTPVEKCTIRPQQNHAVEM
DCDAKIMFDVETFKQDVFVKCHSTPDSVEDVRRNCLKCPILLSPSDPHVVDSVEY
VLNKHNEQLSGHVYEVLEISRGQHKYEPEAFYVEFAIVEVNCTAQEAHDDHHHC
HPNTAGEDHIAFCKATVFRSHASLEKPKHENFESDCVILDVKEGHAHSHLIEHHI
GKYSTSPGQNSTVECVAECPVAFVNKEVPTDISDRHTTPVKGCPGKILHFQL*

>G.blo_HLP-B
MNSLVALVLLGQMIGSTLSHHLQSHVDCNGEDAEKWADMAVHYINEHNLHGY
KQVFNVINEIHVLPRRPRGKIIILELKLLETECHVLDPTPVENCTVRPPHYHAVEG
DCDVKILHDEGVDKVIGAKCHSDPDSVEDVRRNCPKCPILLPLSDPHVVDSVEYV

LNKHNEKLSGHVYEVLEISRGQHKYEPEAFYVEFAIVEVNCTAQEAHDDHHHCH
PNTAGENHIGFCRATVFRSHASLEKPKDEQFESDCVIFDVKEGHAHSHLIEHHIGN
YNTSPGHNNTVLNLAHSHNHTSASHESHSHEHVAEVPVAVAKREVPTNTPHDHT
HPVKLCPGKVHHFKL*

>G.blo_HLP-A
MNSLVALVLLGQIIGSTLSFQLGPNMDCNTKGTKDWADIGVRYINEHKLDGYKN
ALNIIKIFRLLPSDGRSVIVHFKLNLLETKCHVLDPTPVENCAVRQQHNHAVEMD
CNVRIIHDIATFEDEVFVKCKSTPDSVENVRRNCPKCPILLPPNDPHVVDSVEYVL
NKHNEKLSGHVYEVLEISRGQHKYEPEAFYVEFAIVEVNCTAQEARDGHHQCHP
YTAGEDHIAFCRATVFRSHASLEKPKDENFESDCVILDVKEGHAHSHLIQQHIEK
YSTSPGHNSTDEYVVECPVAFVEKEVPTDMSDHDTPPVKGCPGRVLHFQL*

>B.jar_FETUA1
MNSLVALVLLGQIIGSTLSHHLQSHVDCNGEDAEKWAHMAVHYINEHNQHGYK
CALNVINEIRLLPRRPHGTIVFLKLKVLETECHVLDPTPTENCTVRPQHYHAVGG
DCDVKIIHEEGGDKVIGAKCYSDPDSVEDVRRNCPKCPILLNLNDPQVVDSVEYV
LNKHNEKVSGHVYEVLEISRGQHKNEPEAYYVEFAIVEVNCTAQEAHDDHHQC
HPNTAGENHIGFCRATVFRSHASLEKPKDEQFESDCVIFDVKDGHAHSHLIEHHV
GKYSTSPGHNNTVLNLVHSHNHTSASHESHSHEHVTEVPVAVAKREVPKDVPH
DHTHPVKLCPGKVHHFEL*

>B.jar_FETUA2a/BJ46a
MNSLVALVLLGQIIGSTLSSQVRGDLECDEKDAKEWTDTGVRYINEHKLHGYKY
ALNVIKNIVVVPWDGDWVAVFLKLNLLETECHVLDPTPVKNCTVRPQHNHAVE
MDCDVKIMFNVDTFKEDVFAKCHSTPDSVENVRRNCPKCPILLPSNNPQVVDSV
EYVLNKHNEKLSDHVYEVLEISRGQHKYEPEAYYVEFAIVEVNCTAQELHDDHH
HCHPNTAGEDHIAFCRATVFRSHASLEKPKDEQFESDCVILHVKEGHAHSHLIQQ
HVEKDSISPEHNNTALNFVHPHNDTSTSHESHEHLAEVPVAFVKKELPKDISDRH
TTPVKGCPGKVHHFEL*

>B.jar_FETUA3
MNSMVALVLLGQIIGSTLSSQVRGDLPCDDEDSKWWADVGVRYINEHKLHGYK
YALSVIKNIVVVPWDGDWVAVFLKLNLLETECHVLDPTPVKNCTVRTQHNHAV
EMDCDVKIMFNVETFKEDVFAKCHSTPDSVENVRRNCPKCPILLPSNNPQVVDS
VEYVLNKHNEQLSDHVYEVLEISRGQHKYEPEAYYVEFAIVEVNCTAQEAHDDH
HQCHPNTAGEDHIGFCRATVFRSHASLEKPKDEQFESDCVILHVKKGHAHSHLIQ
QHVEKDSISPEHNNTALNFVHPHNDTRASHESHEHLAKVPVAFVKKELPKDISDR
HTTPVVGCPGLRAVGQPY*

>B.jar_FETUA2b
MNSLVALVLLGQIIGSTHSYLVKEEDLNCNSKAAKYRADQAVHYINEHKLHGYK
YALNVIKIFHLLPPEEFSTVFYLKLNLLETECHVFDPTPVENCNVRPQHNHAVEM
DCDVKIIFNFQFFKTEVFVKCNSTPDSVENVRRNSPKCSILLPPNDPQVIDSVEYVL
NKHNEQLSGHVYKVLEISRGQHKYEPEAYYVEFAIMEINCTAQEAHDDHHQCHP
NTAGEDHIGFCRATVFRSHASLEKPKDEQFESDCVILDVKEGHAHSHLIQQHVEK

YSTSPGHNNTALNLVHLHNDTSTSHKSHSGKCPVVFPKKKVPTDIPHHTHPVEGC
PGKVLHFEL*

>C.tig_FETUA1
MNSLVALVLLGQIIGCTFSHHLQSQVDCNGEDAEKWADMAVHYINEHNLHGYK
YTTNVINEIHVLPRRPHGVIVFLELKVLETQCHVLDPTPVENCTVRPQHYHAVGG
DCDVKILHDEGVDKVIGAKCHSDPDSVEDVRQNCSKCPILLSLSDPHVVDSVEY
VLNKYNEKLSGHVYEVLEISRGQHKYEPEAFYVEFAIVEVNCTAEEAHDDHHHC
HPNTDGENHIGFCKATVFRSHASLEKPKDEMFESDCVIFDVKEGHAHSHLIEHHV
GKYSTSPGHNSTVLNLVHSHNHTSASHESHSHEHVMEVPVAVAKREVPIDVPHD
HTHPVKLCPGKVHHFQL*

>C.tig_FETUA2
MNSLVALVLLGXSIGSTLSSQVRGDLECDNKEAKEWADMAVRYINEHKLHGYK
QALNVIKNILVVPWDGDLVAVYLKLNLLETECHMLDPHPVENCTIRPQNNHAVK
MDCNAKIMFDVVTFKQDVFVKCHSTPDSVENVRRNCPKCPILLPWNDSHVVDS
VEYVLNKHNEKLSGHVYEVLEISRGQHKYEPEAFYVEFAIVEVNCTAEEAHDDH
HHCHPNTAGEDHIGFCRATVFRSHASLEKPKDEKFESDCVILHVKEGYAYSHLLY
KQIEKYNVPPEFRNTVLNLTHSHNHTSTSHESHEHVAEVPVAFVKKELPTDISDH
DTPPVEGCPGKALHFQL*

>C.tig_FETUA3
MNSLVALVLLGQIIGSTLSFQLAPNMDCNTKGTKDWADIGVRYINEHKLHGYKQ
ALNIIKIFRLLPSDGLSVMFHFKLNLLETECHVLDPTPVENCIVRQKNNHAVEMD
CNVRIIHDIATFGDEVFVKCNSTPDSVENVRRNCPKCPILLPPNDPHVVDSVEYVL
NKHNEQLSGHVYEVLEISRGQHKYEPEAFYVEFAIVEVNCTAEEAHDDHHHCHP
NTAGEDHIGFCRATVFRSHASLEKPKDEKFESDCVILHVKKGHAYSHLIEHHVGK
YNTSPGRNSTDECVVECPVAFVNKEVPKDISDHDTPPVEGCPGKALHFQL*

>C.tig_FETUA4
MNSLVVLVLLGQIIGSTHSYMVREEHLNCNSKAAKYRADQAVRYINEHKLHGY
KQALNIIKTFHLLPPERFTTVFYLKLNLLETECHVLDPTPVENCTVRPQNNHAVE
MDCDVKINFNFEFFKTEIFVKCNSTPNSVENVRRNCPECPILLPPNDPHVIDSVEY
VLNKHNEKLSGHVYEVLEISRGQHKYEPEAFYVEFAIVEVNCTAEEAHDDHHHC
HPNTAGEDHIGFCRATVFRSHASLEKPKDEKFESDCVILHVKEGHATSHLIEHHT
GKYSTSPGRNSTDECVVECPVAFVKKEVPTHIPHHTHPVEGCPGKVFHFQL*

>C.tig_FETUA5
MNSLVVLVLLGQIIGSTLSYMVREEDLNCNSKAAKYRADQAVHYINEHNLHGY
KQALNIIKTIRLLPSDGLSVIIYLKLNLLETECHVLDPTPVENCTVRQQNNHAVET
DCDVKIIYNIETFKDEFLVKCHSTPGSVENILQDCPKCPILMPLSEPHVTDSVEYVL
NKHNEQLSGHVYEVLEISRGQHKYEPEAFYVEFAIVEVNCTAQEAHDDHHHCHP
NTDGENHIGFCRATVFRSYASLEKPKVEMSESDCVILDVKEGHAHSHLIEHHVGK
YSISPGHNNTVLNLAHSHNHTSASHESHSHEHVTEVPVAVAKREVPTDIPHHHTP
SVDGCPGKVLHFKL*

>C.hor_FETUA5
MNSLVALVLLGQIIGSTHSYMVREEDLNCNSKAAKYRADQAVHYINEHNLHGY
KQALNIIKTIRLLPSDGLSVIMYLKLNLLETECHVLDPTPVENCTVRQQHNHAVET
DCDVKIIYNIETFKDEFLVKCHSTPGSVEKILEDCPKCPLLLSLSDPHVVDSVEYV
LNKHNEKLSGHVYEVLEISRGQHKYEPEAFYVEFAIVEVNCTAQEAHDDHHHCH
PNTAGENHIAFCRATVFRSYASLEKPKDEKFESDCVILHVKEGHTHSHLIEHHIGK
YSTSPGHNSTVLNLAHSHNHTSASHESHSHEHVTEVPVAVAKREDPTDIPHHHTP
SVDGCPGKVLHFKL*

>C.hor_FETUA4
MNSLVVLVLLCQIIGSTYSYMVREEDLNCNSKAAKYRADQAVRYINEHKLHGY
KQALNIIKIFHLLPPERFMTVFYLKLNLLETECHVLDPTPVENCTVRPQNNHAVE
MDCDVKINFNFEFFKTEIFVKCNSTPDSVENVQRNCPECPILLPPNDPHVINSVEY
VLNKHNEKLSGHVYEVLEISRGQHKYEPEAFYVEFAIVEVNCTAEEAHNDHHHC
HPNTAGKDHIGFCRATVFRSHASLEKPKDEMFESDCVIFDVKKGHAHSHLIQQHI
GKYNISPGHNNAVLNLAHSHNDTRASQESHSDECVVECPVAFVKKEVPTHIPHH
THPVEGCPGKVFHFQL*

>C.hor_FETUA3
MNSLVALMLLGQIIGSTLSFQLAANMDCNTKGTKDWADIGVRYINEHKLHGYK
QDLNIIKIFRLLPSDGRSVIFHFKLNLLETECHVLDPTPVENCTVRQQNNHAVEMD
CNVRIIHDIATFEDEVFVKCNSTPDSVENVRRNCPKCPILLPPNDPHVVDSVEYVL
NKHNEKLSGHIYEVLEISRGQHKYEPEAFYVEFAIVEVNCTAEEAHDDHHHCHP
NTDGENHIGFCRSTVFRSHASLEKPKDEKFESDCVILHVKEGHAHSHLIEHHVGK
YSTSPGRNSTDECVVECPVAFVNKEVPKDISDHDTPPVEGCPGKVLHFQL*

>C.hor_FETUA2
MNSLVALVLLGQIIGSTLSSQVRGDLECDDKEAKEWADIAVRYINEHKLHGYKQ
ALNVIKNILVVPWNGDLVAVFLKLNLLETECHVLDPTPVENCTVRPQNNHAVEM
DCDAKIMFDVVTFKTDVFVKCHSTPDSVENVRRNCPKCPILLPRNDSHVVDSVE
YVLNKHNEKLSGHVYEVLEISRGQHKSEPEAFYVEFAIVEVNCTAQEAHDDHHH
CHPNTTGEDHIGFCRATVFRSHASLEKPKDEKFESDCVIFDVKEGHAHSHLIEHH
VGKYSTSPGRNSTDECVVECPVAFVNKELPTDISDHHTTPVKGCPGKVLHFQL*

>C.hor_FETUA1
MNSLVALVLLGQIIGCTFSHHLQSQVDCNGEDAEKWADMAVHYINEHNLHGYK
YTNNVINEIHVLPRRPRGEIVFLELKVLETQCHVLDPTPVENCTVRPLHYHAVGG
DCDVKIIHDEGVDKVIGAKCHSDPDSVEEVRRNCPECPILLSLSDPHVINCVEYVL
NKYNEKLSGHVYEVLEISRGQHKYEPEAFYVEFAIVEVNCTAEEAHDDHHHCHP
NTDGENHIGFCRATVFRSHASLEKPKDEMFESDCVIFDVKEGHAHSHLIEHHVGK
YSTSPGHNNTVLNLIHSHNHTSASHESHSHEHMTEVPVAVAKREVPTDVPHDHI
HPVKLCPGKVHHFKL*

>C.scu_FETUA5
MNSLVALVLLGQIIGSTHSYMVREEDLNCNSKAAKYRADQAVHYINEHNLHGY
KQALNIIKTIRLLPSDGLSVIIYLKLNLLETECHVLDPTPVENCTVRQQNNHAVET

DCDVKIIYNIETFKDEFLVKCHSTPGSVEKILQDCPKCPILLSLSDPHVVDSVEYVL
NKHNEQLSGHVYEVLEISRGQHKYEPEAFYVEFAIVEVNCTAQEAHDDHHHCHP
NTAGEDHIAFCRATVFRSYASLEKPKDEMSESDCVILDVKEGHAHSHLIEHHTGK
YSTSPGHNSTVLNLAHSHNHTSASHESHSHEHVTEVPVAVAKREVPTDIPHHHTP
SVDGCPGKVLHFKL*

>C.scu_FETUA4
MNSLVALVLLGQIIGSTLSYMVREEDLNCNSKAAKYRADQAVRYINEHKLHGY
KQALNIIKTFHLLPPERFTTVFYLKLNLLETECHVLDPTPVENCTVRPQYNHAVE
MDCDVKINFNFEFFKTEIFVKCNSTPDSVENVR*NCPECPILLPPNDPHVIDSVEYV
LNKHNEKLSGHIYEVLEISRGQHKYEPEAFYVEFAIVEVNCTAEEAHDDHHHCHP
NTAGEDHIGFCRATVFRSHASLEKPKDEKFESDCVILHVKKGHAHSHLIQQHIGK
YNISPGHHNTVLNLAHSHNDTRASQESHSDECVVECPVAFVKKEVPTHIPHHTHP
VEGCPGKVHHFKL*

>C.scu_FETUA3
DGLSVIFHFKLNLLETECHVLDPTPLENCTVRQQNNHAVEMDCNVRIIHDIATFE
DEVFVKCNSTPDSVENVRRNCPKCPILLPPNDPHVVDSVEYVLNKHNEKLSGHIY
EVLEISRGQHKYEPEAFYVEFAIVEVNCTAEEAHDDHHHCHPNTDGENHIGFCRA
TVFRSHASLEKPKDEKFESDCVILHVKKGHAYSHLIEHHVGKYNTSPGRNSTDEC
VVECPVAFVNKEVPKDISDHDTPPVEGCPGKALHFQL*

>C.scu_FETUA2
MNSLVALVLLGQIIGSTLSSQVRGDLECDDKEAKEWADMAVRYINEHKLHGYK
QALNVIKNILVVPWDGDLVAVYLKLNLLETECHVLDPTPVENCTIRPQNNHAVK
MDCNAKIMFDVVTFKQDVFVKCHSTPDSVENVRRNCPECPILLPRNDSHVVDSV
EYVLNKHNEKLSGHVYEVLEISRGQHKYEPEAFYVEFAIVEVNCTAQEAHDDHH
HCHPNTAGENHIGFCRATVFRSHASLEKPKDEKFESDCVILHVKEGHAHSHLIEH
HVGKYNTSPGRNSTDECVVESSLLTKATGRPVAFVNKEVPADISDHHTTPVKGC
PGKVLHFQL*

>C.scu_FETUA1
MNSLVALVLLGQIIGCTFSHHLQSQVDCNGEDAEKWADMAVHYINEHNLHGYK
YTTNVINEIHVLPRRPHGEIIFLELKVLETQCHVLDPTPVENCTVRPLHYHAVGGD
CDVKILHDEGVDKVIGAKCHSDPDSVEDVRRNCPNCPILLSLSDPHVVDSVEYVL
NKHNEKLSGHVYEVLEISRGQHKYEPEAFYVEFAIVEVNCTAEEAHDDHHHCHP
NTAGENHIGFCRATVFRSHASLEKPKDEKFESDCVIFDVKEGHAHSHLIEHHTGK
YSTSPGHNNTVLNLVHSHNHTSASHESHSHEHMTEVPVAVAKREVPTDVPHDHT
HPVKLCPGKVHHFKL*

>C.atx_FETUA5
MNSLVALVLLGQIIGSTHSYMVREEDLNCNSKAAKYRADQAVHYINEHNLHGY
KQALNIIKTIRLLPSDGLLVVIYLKLNLLETECHVLDPTPVENCTVRQQNNHAVET
DCDVKIIYNIETFKDEFLVKCHSTPGSVEKILQDCPKCPILLSLSDPHVVDSVEYVL
NKHNEQLSGHVYEVLEISRGQHKYEPEAFYVEFAIVEVNCTAEEAHDDHHHCHP
NTDGENHIGFCRATVFRSHASLEKPKDEMSESDCVILDVKEGHAHSHLIEHHTGK

YSISPGHNNTVLNLAHSHNHTSASHESHSHEHVTEVPVAVAKREVPTDIPHHHTP
SVDGCPGKVLHFKL*

>C.atx_FETUA4
MNSLVVLVLLGQIIGSTHSYMMREEDMNCNSKAAKYRADQAVRYINEHKLHGY
KQALNIIKTFHLLPPERFTTVFYLKLNLLETECHVLDPTPVENCTVRPQNNHAVE
MDCDVKINFNFEFFKTEIFVKCNSTPDSVENVQRNCPKCPILLPPNDPHVINSVEY
MLNKHNEKLSGHVYEVLEISRGQHKYEPEAFYVEFAIVEVNCTAEEAHDDHHHC
RPNTAGEDHIGFCRATVFRSHASLEKPKDEMFESDCVILHVQKGHAHSHLIQQHI
GKYNISPGHHNTVLNLAHSHNDTRASQESHSDECVVECPVAFVKKEVPTHIPHH
THPVEGCPGKVHHFKL*

>C.atx_FETUA3
MNSLVALVLLGQIIGSTLSFQLAGNMDCNTKGTKDWADIGVRYINEHKLHGYKQ
ALNIIKIFRLLPSDGRSVIFHFKLNLLETECHVLDPTPLENCTVRQQNNHAVEMDC
NVRIIHDIATFEDEVFVKCNSTPDSVENVRRNCPKCPILLPPNDPHVVDSVEYVLN
KHNEKLSGHIYEVLEISRGQHKYEPEAFYVEFAIVEVNCTAEEAHDDHHDCHPNT
DGENHIGFCRSTVFRSHASLEKPKDEKFESDCVILHVKKGHAYSHLIEHHVGKYN
TSPGRNSTDECVVQCPVAFVNKEVPKDISDHDTPPVEGCPGKALHFQL*

>C.atx_FETUA2
MNSLVALVFLGQIIGSTLSSQVRGDLECDDKEAKEWADMAVRYINEHKLHGYK
EALNVIKNILVVPWDGDLVAVFLKLNLLETECHVLDPTPVENCTVRPQNNQAVE
MDCDAKIMFDVVTFKKDVFVKCHSTPDSVENVRRNCPKCPILLPRNDSHVVDSV
EYVLNKHNEKLSGHIYEVLEISRGQHKYEPEAFYVEFAIVEVNCTAQEAHDDHH
HCHPNTAGEDHIGFCRATVFRSHASLEKPKDEKFESDCVILHVQEGHAHSHLIEH
HTGKYSTSPGSNSTDECVVQCPVAFVNKELPTDISDHHTTPVKGCPGKVFHFQL*

>C.atx_FETUA1
MNSLVALVLLGQIIGCTFSHHLQSQIDCNGEDAEKWADMAVHYINEHNLHGYK
YTTNVINEIHVLPWRPHGEIIFLELKVLETQCHVLDPTPVENCTVRPLHYHAVGG
DCDVKIIHDEGVDKVIGAKCHSDPDSVENVRQNCPECPILLSLSDPHVVDCVEYV
LNKYNEKLSGHIYEVLEISRGQHKYEPEAFYVEFAIVEVNCTAEEAHDDHHHCHP
NTDGENHIGFCRATVFRSHASLEKPKDEMFESDCVIFDVKEGHAHSHLIEHHTGK
YSTSPGHNNTVLNLIHSHNHTSASHESHSDEHMTEVPVAVAKREVPTDVPHDHT
HPVKLCPGKVHHFKL*

>C.ada_FETUA5
MNSLVALVLLGQIIGSIHSYMVREEDLNCNSKAAKYRADQAVQYINEHNLHGYK
QALNIIKTIRLLPSDGLLVVIYLKLNLLETECHVLDPTPVENCTVRPQNNHAVETD
CDVKIIYNIETFKDEFLVKCHSTPGSVEKILQDCPKCPILLPPNDPHVVDVEYVLN
KHNEKLSGHVYEVLEISRGQHKYEPEAFYVEFAIVEVNCTAQEAHDDHHHCHPN
IAGEDHIGFCRATVFRSYASLEKPKDEKFESDCVILDVKEGHAHSHLIQQHIGKYS
TSPGHNNTVLNLAHSHNHTSASHESHSHEHVTEVPVAVAKREDPTDIPHHHTPSV
DGCPGKVLHFKL*

>C.ada_FETUA4
MNSLVVLVLLGQIIGSTHSYMVREEDMNCNSKAAKYRADQAVRYINEHKLHGY
KQALNIIKTFHLLPPERFTTVFYLKLNLLETECHVLDPTPVENCTVRPQNNHAVE
MDCDVKINFNFEFFKTEIFVKCNSTPDSVENVRRNCPECPILLPPNDPHVIDSVEY
VLNKHNEKLSGHIYEVLEISRGQHKYEPEAFYVEFAIVEVNCTAEEAHDDHHHC
HPNTDGENHIGFCRATVFRSHASLEKPKDEKFESDCVILHVKEGHAHSHLIQQHI
GKYNISPGHNNTVLNLAHSHNDTRASQESHSDECVVECPVVFVKKEVPTHIPHH
THPVEGCPGKVFHFQL*

>C.ada_FETUA3
MNSLVALMLLGQIIGSTLSFQLAGNMDCNTKGTKDWADIGVRYINEHKLHGYK
QALNIIKIFRLLPSGGLSVIFHFKLNLLETECHVLDPTPVENCTVRPQNNHAVEMD
CNVRIIHDIATFEDEVFVKCNSTPDSLENVRRNCPKCPILLPPNDPHVVDSVEYVL
NKHNEKLSGHIYEVLEISRGQHKYEPEAFYVEFAIVEVNCTAEEAHDDHHHCHP
NTDGENHIGFCRATVFRSHASLEKPKDEKFESDCVILHVKEGHAHSHLIQHHTGK
YSTSPGRNSTDECVVECPVAFVNKELPTDISDHDTPPVEGCPGKVLHFQL*

>C.ada_FETUA2
MNSLVALVFLGQIIGSTLSSQVRGDLECDDKEAKEWADMAVRYINEHKLHGYK
EALNVIKNILVVPWNGDLVAVFLKLNLLETECHVLDPTPVENCTVRPQNNHAVE
MDCDAKIMFDVVTFKKDVFVKCLSTPDSVENVRRNCPKCPILLPRNDSHVVDSV
EYVLNKHNEKLSGHVYEVLEISRGQHKYEPEAFYVEFAIVEVNCTAQEAHDDHH
HCHPNTAGEDHIGFCRATVFRSHASLEKPKDEKFESDCVILHVKEGHAHSHLIEH
HTGKYSTSPGRNSTDECVVQCPVAFVNKELPTDISDHHTTPVKGCPGKVFHFQL*

>C.ada_FETUA1
MNSLVALVLLGQIIGCTFSHHLQSQIDCNGEDAEKWADMAVHYINEHNLHGYK
YTTNVINEIHVLPRRPHGEIIFLELKVLETQCHVLDPTPVENCTVRPLHYHAVGGD
CDVKIIHDEGVDKVIGAKCHSDPDSVENVRQNCPECPILLSLSDPHVIDSVEYVLN
KYNEKLSGHVYEVLEISRGQHKYEPEAFYVEFAIVEVNCTAEEAHDDHHHCHPN
TDGENHIGFCRATVFRSHATLEKPKDEMFELDCVIFDVKEGHAHSHLIEHHVGKY
STSPGHNNTVLNLIHSHNHTSASHESHSHEHMMEVPVAVAKREVPTDVPHDHTH
PVKLCPGKVHHFKL*

>Drus_FETUA1
MNSLVALLLLGQIIGCTFSHHLPSHGDCNGEDAKKWANLAVDYINEHTLHGYKQ
ELNIIKDIHVLPRRPHGKIAFLKLELLETECHVLDKTPVKNCTVRPQQNHAVEGEC
DVKIIHDEDVDKVVAVKCHSSPDSVEDVRRNCPNCPILLRLNDPRVVDAVEYVL
NKVNEKLPSYIYELLEITRGQLTFEPEGFYVEFAIVQSNCTAQEAQDDHHQCHPN
TEEEDHTGFCKATLFRSHDSLGKPKDEQFQPDCELFDVKEGLAYSHLIEHRDGKY
TSPGYNSTVLNLAHSHNHTSASQESHSHEHVVEVAVAVAKREIPTDIPPHHTDPV
NLCPGEVYHFTL*

>Drus_FETUA4/5
MNSLVALLLLSQIIGSTLNYIVTKKHLNCNSSGATGKANRAVHYINEHNRHGYK
QALNEIEIIRLIPSGGRIVTFYLKLNLLETVCHVLDPTPVEKCTVRPQHYHAVESDC

DVKVVFNYVTFEEEFFVKCNSIPDSVENVQRNCPKCPILLPLNDPHVVDAVEYVL
NKHNEKLSGHVYEVLEISRGQHKFEPEGIYVEFAIVQSNCTAQEAQDDHHHCHP
NTARKDHIGFCRATVFRSNASLEKPKDEEYESDCVIFDVKKGRAHSHLIDHHIGK
YSTSPGDNSTVLNLAHSHNDTRGSPQSHSEQPVDGCPGEVHHFKL*

>Drus_FETUA3
MNSLVVLLLLGQIIGSTLSFQLRGDMDCNSKGTKDWANIGVSYINDHKLHGYKQ
ALNIIKFFDLLPSGGISETFYLKLNLLETECHVLDPTPVEKCTVRPQHNHTVEMDC
NVIIIYNIETFRKTVFVKCNSTTDSVENVQRNCPKCPILLSRNDPDVIDAVEYVLN
KNNEKLSGHVYEVLEISRGQHTFEPEGIYVEFAIVQSNCTAQEAQDDHHHCYPNT
AGEDHIGFCRATVFRSHASLGKPKDEEYEADCEIFDVKKGHAYSHLIKHHVAKY
STSPGDNTTVLNLAHSHNHARASQESHSEECVVECPVAFVKKEVPTYISDHDIPP
VVEGCPGRALHFNL*

>Drus_FETUA2a
MNSLVALLLLSQIIGSTLSSQVRGDIDCNDKDAKDWAGIAVRYINEHKLHGYKQ
ALNVINNIRILPWNGDLVAIYLKLIFLETECHVLDPTPVKNCTVRPRDNHAVKIDC
DVKIIYNIETHKDNVFVKCNSTPDSVENVQRNCPKCPILLPRNDPHVVDAVEYVL
NKHNEKLSGHVYEVLEISRGQHKFEPEGIYVEFAIVQSNCTAQEAQDDHHHCHP
NTAGEAYTGFCRATVFRSHASLGKPKDEEYESDCEIFDVKKGHAHCYLIEHHVG
KYSTSPGDNTTVLNLAHLHNHARASHESHSEECVVGCPVAFVKKEVPTDISDHH
TPPVKGCPGRVLHFNL*

>Cgas_FETUA1
MNSLVALLLVGQIIGSTLSHHLPSHGDCNGEDAEKWAHLAVHYINEHNLHGYKQ
DLNIIKDIHVLPRRPHGKIVFLKLELLETVCHVLDPTPVKNCTVRPQHYHAVEGD
CDVKILHDENEDNVVAVKCHSSPDSVEDVQRNCPNCPILLRLNDPHVVDAVEYV
LNKHNEKLSGHVYEVLEISRGQHKFEPEGYYVEFAIVQSNCTAQEAQDDHHHCH
PNTAGEDHIGFCRATVFRSHASLEKPKDEQFESDCVIFNVKEGHAHSHLIEHHVG
KYSTSPGHNNTVLNLAHSHNHTSASHESHSHEVPVAVAKREVPTNISPPHTHPVN
LCPGKVHHFKV*

>Cgas_FETUA4/5
MNSLVALLLLSQIIGSTLSYIVTKQHLNCNSSGAKGKADRAVHYINEHNLHGYKQ
ALNEIEIIRLIPSGGRTVIFYLKLNLLETECHALDPTPVEKCTVRSQHNHAVESNCD
VKVIYNYVTYQEQFFVKCNSIPDSVENVQRNCSKCPILLPLNDPHVVDAVEYVLN
KHNEKLSGHVYEVLEISRGQQKFEPEGYYVEFAIVQSNCTAQEAQDDHHHCHPN
TAGEDHIGFCRATVFRSHANLEKPKDEQFESDCVILGVKYGHAHAHLIDHHIGKY
STSPGDNSTVLNLVHSHNDTRASPEFHSSEKTPVDGCPGKVRHFKL*

>Cgas_FETUA3
MNALVALLLLGQIIGSTLSFQLVGNMDCNTKGTKDWADIGVRYINDHKLHGYK
QALNRIKIFRLLPSGGIVETFYFKLNFLETECHVLDPTPVEKCTVRPQHNHTVEMD
CDVIIIYNIDTFGKEVFAKCNSTADSVENVQRNCPKCPILLPRNDPHVVDAVEYVL
NKHNEKLSGHVYEVLEISRGQQKFEPEGYYVEFAIVQSNCTAQEAQDDHHHCHP
NTAGEDHIGFCRATVFRSHASLEKPKDEQFESDCVIFDVKMGHAYSHLIEQHVG

KYSTSPGDNTTVLNLAHSHNHTSDSQESHSDECMVGCPVAFVKKEVPTDISDHD
TPPVKGCPGRVLHFHL*

>Cgas_FETUA2a
MNFLVALLLLSQIIGSALSSQISGLVYCNDKEAKDWADKAVRYINEHKLHGYKQ
ALNVIKSIRILPWDGDLVAIYLKLNFVETECHVLDATPVENCIVRPRDNHTVETDC
DVKIIYNIETYKDEVFVKCISTPDSVENLQRICPKCPILLPRNDPHVVDAVEYVLN
KHNEKLSGHVYEVLEISRGQHKFEPEGYYVEFAIVQSNCTAQEAQDDHHHCHPN
TAGEDHIGFCRATVFRSHASLEKPKDEQFESDCVIFDVKKGHAYSHLIEQHVGKY
STSSGDNTTVLNLAHSHNHTRDSQESHSDECVVGCPVAFVKKEVPTDISDHHTPP
VKDCPGRVLHLNP*

>Cgas_FETUA2b
MNSLVALLLLGQIIGSTLSSQVRGDIDCNDKDAKEWADMAVRYINEHKLHGYKL
VLNVIKNIHILTWSRDLVAGYLKLNLLETECHVLDPTPVKNCTVRPQHNHAVET
DCDVKVIYNVMTLKDEVFVKCNSTPDSVENVQRNCPKCPILLPRNDPHVVDAVE
YVLNKHNEKLSGHVYEVLEISRGQHKFEPEGYYVEFAIVQSNCTAQEAQDDHHH
CHPNTAGEDHIGFCRATVFRSHASLEKPRHEQFESDCVIFDVKMGHAYSHLIKQH
VGKYSTSPGDNTTVLNLAHSHNHTRDSQESHSDECMVGCPVAFVKKEVPTDISD
HHTPPVKGCPGRVLHFHL*

>Msch_FETUA1
MNSLVALLLLGQIIGCTFSHHLPSHGDCNGEDAKKWAHLAVHYINEHNLHGYK
QDLNIIKDIHVLPRRPHGKIVFLELKLLETVCHVLDPTPVENCTVRPQHYHVAVE
GDCDVKIIHDEDVDKVVAAKCHSSPDSVEDVRRNCPNCPILLRLNDPHVVDTVE
YVLNKHNEKLSGHVYEVLEISRGQHTFEPEGFYVEFAIVQSNCTAQEAQDDHHH
CHPNTAGEDHIGFCRATVIRSHASLEKPKDEQFESDCVIFDVKEGHSYSHLIEHHV
GKYSTSPGHNNTVLNLAHSHNHTSASHESHSHEHVVEVPVAVAKREIPTDIPPHH
THPVNLCPGKVHHFKV*

>Msch_FETUA3
MNSLVALLLLGQIIGSTLSFQLAGDMDCNTKGTKDWANIGVRYINDHKLHGYKQ
ALNIIKIFRLLPSDGISETFYFKLNLLETECHVLDPTPVEKCTVRPQHNHTVEMDC
NVIIIYNIETFREKVFVKCNSTTDSVESVQRNCPKCPILLPRNDPHVVDAVEYVLN
KHNEKLSGHVYEVLEISRGQHKFEPEGFYVEFAIVQSNCTAQEAQDDHHHCHPN
TAGEDHIGFCRATVFRSHASLEKPKDEQFESDCVIFDVKNGHAYSHLIKHHIGKY
STSPGDNTTVLNLAHSHNHTRASQESHSDECVVGCPVAFVKKEVPTYLSDHDTP
PVKGCPGRVLHFKL*

>Msch_FETUA2a
MNSVVALLLLSQIIGSTLSSQVRGDIDCNDKDAKDWAGKAVRYINEHKLHGYKQ
ALNVIKNIRILPWDGDLVAIYLKLNFLETECHVLDPTPVENCTVRPRDNHAVKID
CDVKIIYDIETHKDNVFVKCNSTPDSVENVQQNCPKCPILLPRNDPHVVDAVEYV
LNKHNEKLSGHVYEVLEISRGQHKFEPEGFYVEFAIVQSNCTAQEAQDDHHHCH
PNTAGEDHIGFCRATVFRSHASLEKPKDEQFESDCVIFDVKKGHAHCYLIEHHVG

KYSTSPGDNTTVLNLAHSHNHTRASHESHSDECVVGCPVAFVKKEVPTDISDHD
TPPVKGCPGRVLHFNL*

>Msch_FETUA2b
MNSLVALLLLGQIIGSTLSSQVRGDIDCNNKDAKEWADIAVRYINEHKLHGYKQ
VLNVIKNIRIVTWARDLVASYLKLNLLETECHVLDPTPVEKCTVRPQHNHTVETD
CDVKVIYNVMTLKDEVFVKCNSTPDSVENVQRNCPKCPILLPRNDPHVIDAVEY
VLNKHNEKLSDHVYEVLEISRGQHTFEPEGFYVEFAIVQSNCTAQEAQDDHHHC
HPNTAGEAHIGFCRATVFRSHATLEKPKDEEFESDCVIFDVKKGHAHSHLIEQQIG
KYSTSPGHNNTVLNLAHSHNDTRASHESHSDECVVECPVTFVKKEVPTDISDHH
TPPVKGCPGRVHHFKL*
>Vseo_FETUA1
MNSLVALLLLGQIIGCTFSHHLPSHGDCNGEDAKKWAHLAVHYINKHTLHGYK
QDLNIIKDIHVLPRRPHGKIVFLELELLETVCHVLDPTPVENCTVRPQHYHAVEGD
CDVKIIHDEDVDNVVAAKCHSSPDSVEDVRQNCPKCPILLPLNDPHVVDAVEYV
LNKHNEKLSGHVYEVLEISRGQHTFEPEGFYVEFAIVESNCTDQEAQDDHHHCHP
NTAGEDHIGFCRANVIRSHASLEKPKDEQFESDCVIFDVKEGHSYSHLIEHHVGK
YSTSPGHNNTVLNLAHSHNHTSASHESHSHEHVVEVPVAVAKREIPTNIPPHHTH
PVNLCPGKVHHFKV*

>Vseo_FETUA2a
MNSLVALLLLSQIIGSTLSSQVRGDIDCNDKDAKDWAGKAVRYINEHKLHGYKQ
ALNVIKNIHILPWDGDLVAIYLKLNFLETECHVLDPTPVENCTVRPQHNHAVKID
CDVKIIYDIETHKDDVFVKCNSTPDSVENVQQNCPKCPILLPLNDPHVVDAVEYV
LNKHNEKLSGHVYEVLEISRGQHKFEPEGFYVEFAIVESNCTAQEAQDDHHHCH
PNTEGEDHIGFCRATVFRSHASLEKPKDEQFESDCVIFDVKKGHAHCYLIEQHVG
KDSTSPGDNTNVLNLAHSHNHTRASHESHSDECVVGCPVAFVKKEVPTDISDHD
TPSVKGCPGRVLHFNL*

>Vseo_FETUA2b
MNSLVALLFLGQVIGSTLSSQVRGDIDCNDKDAKHWADIAVRYINEHKLHGYKQ
DLNVIKNIHILTWDRDLVAGYLKLNLLETECHVLDPTPVEKCTVRPQHNHTVEM
DCDVKIIYNVMTLKDEVSVKCNSTTDSVENVQRNCPKCPILLPRNDPHVVDAVE
YVLNKHNEKLSGHVYEVLEISRGQHTFEPEGFYVEFAIVQSNCTAQEAQDDHHH
CHPNTAGEDHIGFCRATVFRSHASLEKPKDEQFESDCVMFDVKEGHTHSYLIEQH
IGKDSTSPGDNTTVLNLAHSHNHTRTSHEFHSDECVVECPVAFIKKEVPTDISDHH
TPPVKGCPGRVLHFKL*

>Vamm_FETUA1
MNSLIALLLLGQIIGCTFSHHLPSHGDCNGEDAKKWAHLAVHYINEHTLHGYKQ
DLNIIKDIHVLPRRPRGKIIFLELELLETVCHVLDPTPVENCTVRPQHYHAVEGDC
DVKIIHDEDVDKVVAAKCHSSPDSVEDVRRNCPKCPILLPLNDPHVVDAIKYVLN
KHNEKLSGHVYEVLEISRGQYTFEPEGFYVEFAIVQSNCTAQEAQDDHHHCHPN
TAGEDHIGFCRANVIRSHASLEKPKDEQFESDCVIFDVKEGHSYSHLIEHHVGKY
STSPGHNNTVLNLAHSHNHTSASHESHSHEHVVEVPVAVAKREIPTNIPPHHTHP
VNLCPGKVHHFKV*

>Vamm_FETUA2a
MNFVVALLLLSQIIGSTLSSQVRGDIDCNDKDAKDWAGKAVRYINEHKLHGYKQ
DLNVIKNIHILPWNGDLVAIYLKLNFLETECHVLDPTPLENCTVRPRDNHAVKMD
CDVKMIYDIETHKNDVFVKCNSTPDSVEHVQQNCPKCPILLPQNDPHVVDAVEY
VLNKHNEKLSGHVYEVLEISRGQHTFEPEGFYVEFAIVESNCTAQEAQDDHHHC
HPNTEGEDHIGFCRATVIRSHASLEKPKDEQFESDCVIFDVKKGHAHCYLIEQHV
GKDSTSPGDNTTVLNLAHSHNHTRASHESHSDECVVGCPVAFVKKEVPTDISDH
DTPSVKGCPGRVLHFNL*

>Vamm_FETUA2b
MNSLVALLLLGQIIGSTLSSQVRGDIDCNDKDAKHWADIAVRYINEHKLHGYKQ
DLNVIKNIHILTWDRDLVAGYLKLNLLETECHVLDPTPVEKCTVRPQHNHTVEM
DCDVKIIYNVMTLKDEVSVKCNSTTDSVENVQRNCPKCPILLPRNDPHVVDAVE
YVLNKHNEKLSGHVYEVLEISRGQHTFEPEGFYVEFAIVQSNCTAQEAQDDHHH
CHPNTEGEDHIGFCRATVFRSHASLEKPKDEQFESDCVMFDVKEGHAHSYLIEQH
VGKDSTSPGDNTTVLNLAHSHNHTRASHEFHSDECVVECPVAFVKKEVPTDISD
HHTPPAKGCPGRVLHFKL*

>Vber_FETUA1
MNSLIALLLLGQIIGCTFSHHLPSHGDCNGEDAKKWAHLAVHYINEHTLHGYKQ
DLNIIKDIHVLPRRPRGKIIFLELELLETVCHVLDPTPLENCTVRPQHYHAVEGDC
DVKIIHDEDVDKVVAAKCHSSPDSVEDVRQNCPKCPILFPLNDPHVVDAVEYVL
NKHNEKLSGHVYEVLEISRGQYTFEPEGFYVEFAIVESNCTAQEAQDDHHHCHP
NTAGEEHIGFCRANVVRSHASLEKLKDEQFESDCVIFDVKEGHSYSHLIEHHIGK
YSTSPGHNNTVLNLAHSHNHTSASHESHSHEHVVEVPVAVAKREIPTNIPPHHTH
PVNLCPGKVHHFKV*

>Vber_FETUA2a
MNSLVALLLLSQIIGSTLSSQVRGDIDCNDKDAKDWAGKAVRYINEHKLHGYKQ
DLNVIKNIHILPWDGDLVAIYLKLNFLETECHVLDPTPLENCTVRPQHNHAVKMD
CDVKMIYDIETHKNDVFVKCNSTPDSVENVRQNCPKCPILLPLNDPHVVDAVEY
VLNKHNEKLSGHVYEVLEISRGQHTFEPEGFYVEFAIVESNCTAQEAQDDHHHC
HPNTAGEEHIGFCRATVIRSHASLEKPKDEQFESDCVIFDVKKEHAHCYLIEQHV
GKDSTSPGDNTNVLNLAHSHNHTRASHESHSDECVVGCPVAFVKKEVPTDISDH
DTPSVKGCPGRVLHFNL*

>Vber_FETUA2b
MNSLVALLLLGQVIGSTLSSQVRGDIDCNDKDAKHWADIAVRYINEHKLHGYKQ
DLNVIKNIHILTWDRDLVAGYLKLNLLETECHVLDPTPVEKCTVRPQHNHTVEM
DCDVKIIYNVMTLKDEVSVKCNSTTDSVENVQRNCPKCPILLPRNDPHVVDAVE
YVLNKHNEKLSGHVYEVLEISRGQHTFEPEGFYVEFAIVQSNCTAQEAQDDHHH
CHPNTAGEDHIGFCRATVFRSHASLEKPKDEQFESDCVMFDVKEGHTHSYLIEQH
VGKDSTSPGDNTTVLNLAHSHNHTRASHEFHSDECVVECPVAFVKKEVPTDISD
HHTPPAKGCPGRVLHFKL*

>Vlat_FETUA1
MNSLVALLLLGQIIGCTFSHHLPSHGDCNGEDAKKWAHLAVHYINKHTLHGYK
QDLNIIKDIHVLPRRPHGKIVFLELELLETVCHVLDPTPVENCTVRPQHYHAVEGD
CDVKIIHDEDVDNVVAAKCHSSPDSVEDVQQNCPTCPILLPLNDPHVVDAVEYV
LNKHNEKLSGHVYEVLEISRGQHTFEPEGFYVEFAIVESNCTAQEAQNDHHHCHP
NTAGEDHIGFCRANVIRSHASLEKPKDEQFESDCVIFDVKEGHSYSHLIEHHVGK
YSTSPGHNNTVLNLAHSHNHTSASHESHSHEHVVEVPVAVAKREIPTNIPPHHTH
PVNLCPGKVHHFKV*

>Vlat_FETUA2a
MNSLVALLLLSQIIGSTLSSQVRGDIDCNDKDAKDWAGKAVRYINEHKLHGYKQ
ALNVIKNIHILPWDGDLVAIYLKLNFLETECHMLDPTPVENCTVRPQHNHAVKID
CDVKIIYDIETHKDDVFVKCNSTPDSVENVQQNCPKCPILLPQNDPHVVDAVEYV
LNKHNEKLSGHVYEVLEISRGQHTVFSFCIRMAVMMVILGFLSSAVTLHNSKLHI
ETFRLKHIGFCRATVFRSHASLEKPKDEQYESDCVIFDVKGHAHCYLIEQHVGKD
STSPGDNTNVLNLAHSHNHTRASHESHSDECVVGCPVAFVKKEVPTDISDHDTPS
VKGCPGRVLHFNL*

>Vlat_FETUA2b
MNSLVALLLLGQVIGSTLSSQVRGDIDCNDKDAKHWADIAVRYINEHKLHGYKQ
DLNVIKNIHILTWARDLVAGYLKLNLLETECHVLDPTPVEKCTVRPQHNHTVEM
DCDVKIIYNVMTLKDEVFVKCNSTTDSVENVQRNCPKCPILLPRNDPHVVDAVE
YVLNKHNEKLSGHVYEVLEISRGQHTFEPEGFYVEFAIVQSNCTAQEAQDDHHH
CHPNTAGEDHIGFCRATVFRSHASLEKPKDEQFESDCVMFDVKEGHTHSYLIEQH
IGKDSTSPGDNTTVLNLAHSHNHTRASHEFHSDECVVECPVAFVKKEVPTDISDH
HTPPAKGCPGRVLHFKL*

>Afea_FETUA1
MNSLVALVLLAQIIGSTLSHHLPSHVDCNGEDAEKWADMAVHYINEHNVHGYK
QALNVIKEIRVLPRRPRGEIVYLELELLETVCHVLDPTPVENCTVRPQHYHAVEG
DCDVKIIHDEGVDKVVGAKCHSNPDSLEDVRQNCLKCPILLPLSDPHVVDSVEY
VLNKHNEKLSDHVYEVLEISRGQHKYEPEAFYVEFAIVETNCTAQESHDDHHHC
HPNTAGEAHIGFCRATVFRSHASLEKPKDEKIESDCVIFDVKEGHAHSHLIEHHV
GKYSVSPGHNSIVLNLAHSHNHSSASHESHSHEHVTEVPVAVAKREVPTDIPHHH
THPVKGCPGKVHHFKL*

>Afea_FETUA2
MNSLVALVLLGQIIGSTLSSQVRGDLECDDKEAKEWAADAVRYINEHKLRGYKQ
ALNVIKNILVVPWNGDLVAIFLKLNLLETECHVLDPTPVENCTVRPQHNHTVEM
DCDAKIIFDIVTFKQDVFVKCNSTPDSLENVRRNCPKCPILLPLNDPHVVDSAEYV
LNKHNEKLSDHVYEVLEISRGQHKYEPEAFYVEFAIVETNCTAQESHDDHHHCH
PNTAGEDHIAFCRATVFRSHASLEKPKDEKFESDCVIFDVKEGHAHSHLIEQHES
HSDECVAEALFAFVKKERPTDISDRHTHPVKGCPGKVHHFEL*

>Afea_FETUA3

MNSLVALVLLGHIIGSTLSFQLPGDMDCNTKGTKDWADIGVRYINEHKLHGYKQ
ALNVIKIFRLLPSDGRSVIFYFKLNLLETECHVLDPTPVEKCTVRPQHNHAVEMD
CNVKIIYDIVTFKDEVFVKCNSTPDSLENVRRNCPKCPILLPLSDPHVVDSAEYVL
NKHNEKLSDHVYEVLEISRGQHKYEPEAFYVEFAIVETNCTAQESQDDHHHCHP
NTAGEDHIGFCRATVFRSHASLEKPKDEKVESDCVIFDVKEGHAHSYLIEQHVGK
YSVSSGHNSTVLNLAHSHNHTRPSHESHSDECVMECPVAFVKKEVPTDISDHDTP
PVEGCPGRVLHFKL*

>Balt_FETUA1
MNSLVALVLLGQIIGSTLSHHLQSHVDCNGKDAEKWADMAVHYINEHNQHGYK
CALNVINEIRLLPRRPHGTIVFLELKVLETVCHVLDPTPIENCTVRPQHYHAVGGD
CDVKIIHEEGGDKVIGAKCYSDPDSVEDVRRNCPKCPILLNLNDPQVVDSVEYVL
NKHNEKVSGHVYEVLEISRGQHKYEPEAYYVEFAIVEVNCTAQELHDDHHQCHP
NTAGENHIGFCRGTVFRSHASLEKPKDEQFESDCVIFDVKDGHAHSHLIEHHVGK
YSTSPGHNNTVLNLVHSHNHTSASHESHSHEHVAEVPVAVAKREVPKDIPHDHT
HPVKLCPGKVHHFEL*

>Balt_FETUA2p
MNSLVALVLLGQIIGSTLSSQVRGDLECDEKDAKEWTDIGVRYINEHKLHGYKY
ALNVIKNIVVVPWDGDWVAVFLKLNLLETECHVLDPTPVKNCTVRTQHNHAVE
MDCDVKIMFNVDTFKEDVFAKCHSTPDSVEDVRRNCPKCPILLPPNNPQVVDSV
EYVLNKHNEKLSDHVYEVLEISRGQHKYEPEAYYVEFAIVEVNCTAQELHDDHH
QCHPNTAGEDHIGLCRATVFRSHASLEKPKDEQFESDCVILHVKEGHAHSHLIQQ
HVEKESISPEHSNTAVNLVHPHNDTNTSHESHEHLAEVPVAFVKKELPKDISDRH
TTPVKGCPGKVLHFQL*

>Balt_FETUA2q
MNSLVALVLLGQIIGSTLSSQVRGDLPCDDKDSKWWADIGVRYINEHKLHGYKY
ALNVIKNIVVTSRDRDRVAVFLKLNLLETECHVLDPTPVKNCAVRTQHNHAVEM
DCDVNIMFNIETFKKDVFVKCHSTPDSVENVRRNCTKCPILLPPNNPQVVDSVEY
VLNKHNEKVSGHVYEVLEISRGQHKYEPEAYYVEFAIVEVNCTAQEAHDDHHH
CHPISAGEDHIAFCRATVFRSHASLEKPKDEQVESDCVILHVKKGHAHSHLIQQH
VEKDSISPEHNNTALNLVHPHNDTSTSHESHEHLAEVPVAFVKKELSKDISDRHT
TPVKGCPGKVLHFQL*

>Balt_FETUA2r
MNSLVALVLLGQIIGSTLSSQVRGDLQCDDEDAKEWTDRGVRYINEHKLHGYKY
ALNVIKNIVVVPLDGDWVAVFLKLNLLETECHVLDPNPFDNCTVRPQHNHAVE
MDCDVKIMFNVDTFKEDVFAKCHSTPDSVENMQRNCPKCPILLPSNDPQVVDSA
KYLLSKDNEKLFDQVYLYELLDISRGQHKYEPEAYYVEFAIVEVNCTAHELHDD
HHQCHPNTAGEDHVALCIGTVFRSNASLEKLKDEKFEVDCFTILAKKGHAYSHLI
QQLFEKKIISPEHNNTALNLIHPHNDTSTSHESHEHVAEVPVAFVKKELPKDISDH
HTTPVKGCPGIVLHFEL*

>Balt_FETUA2s

MNSLVALVLLGQIIGSTLSSQVRGDLPCDDKDSKWWADIGVRYINEHKLHGYKY
ALNVIKNIVVTSRDHDRVAVFLKLNLLETECHVLDPTPVKNCAVRTQHNHAVEM
DCDVNIMFNIETYKKDVFVKCHSTPDSVEDVRRNCTKCPILLPSNNPQVVDSVEY
VLNKHNEQLSGHVYELLDISRGQHKYEPEAYYVEFAIVEVNCTAQEAHDDHHH
CHPISAGEDHIAFCRATVFRSHASLEKPKDEQVESDCVILHVKKGHAHSHLIQQH
VEKDSISPEHNNTALNLVHPHNDTSTSHESHEHLAEVPVAFVKKELSKDRSDRHT
TPVKGCPGKVLHFQL*

>Bcot_FETUA1
MNSLVALVLLGQIIGSTLSHHLQSHVDCNGEDAEKWADMAVHYINEHNQHGYK
CALNVINEIRLLPRRPHGTIVFLELKVLETVCHVLDPTPIENCTVRPQHYHAVGGD
CDVKIIHEEGGDKVIGAKCYSDPDSVEDVRRNCPKCPILLSLNDPQVVDSVEYVL
NKHNEKVSGHVYEVLEISRGQHKYEPEAYYVEFAIVEVNCTAQEAHDDHHQCH
PNTAGENHIGFCRGTVFRSHASLEKPKDEQFESDCVIFDVKDGHAHSHLIEHHVG
KYSTSPGHNNTVLNLVHSHNHTSASHESHSHEHVAEVPVAVAKREVPKDIPHDH
THPVKLCPGKVHHFEL*

>Bcot_FETUA2w
MNSLVALVLLGQIIGSTLSSQVRGDLECDEKDAKEWTDVGVRYINEHKLHGYKY
ALNVIKNIVVVPWDGDWVAVFLKLNLLETECHVLDPTPVKNCTVRTQHNHAVE
MDCDVKIMFNVDTFKEDVFAKCHSTPDSVENVRRNCPKCPILLPSNNPQVVDSV
EYVLNKHNEKLSGHVYEVLEISRGQHKYEPEAYYVEFAIVEVNCTAQEAHNDHH
QCHPNTAGENHIAFCRATVFRSHASLEKPKDEQFESDCVILHVKQGHAHSHLIQQ
HVEKDSISPEHNNTALNLIHPHNDTSTSHESHEHVAEVPVAFVKKELPKDISDRHT
TPVKGCPGKVLHFGL*

>Bcot_FETUA2x
MNSLVALVLLGQIIGSTLSSQVREDLECNDETAKWWTDIGVRYINEHKLHGYKY
TLNVIKNIVVTSRDRDRVAVFLKLNLLETECHVLDPTPVKNCAVRTQHNHAVEM
DCDVNIMFNVATFKEDVFAKCHSTPDSVENVRRNCTKCPILLPSNNPQVVDSVE
YVLNKHNEQISGHVYEVLEISRGQHKYEPEAYYVEFAIVEVNCTAQEAHDDHHH
CHPISAGEDHIAFCRATVFRSHASLEKPKDEQVESDCVILHVKKGHAHSHLIQQH
VEKDSISPEHNNTALNLVHPHNDTSTSHESHEHLAEVPVAFVKKELSKDISDRHT
TPVKGCPGKVLHFQL*

>Bcot_FETUA2y
MNFLVALVLLGQIIGSTLSSQVMGDLSCNDEDSRWWADVGVRYINEHKLHGYK
YALSVIKNIIVVPWDGDWVAVFLKLNLLETKCHVLDPTPVKNCNVRTQHNHAV
EMDCDVKIMFNVATFKEDVFAKCHSTPDSVEDVRRNCPKCSILLPSNHPQVVDS
AEYVLNKHNEKLSDHVYEVLEISRGQHKYELEAYYVEFAIVEVNCTAQEAHDD
HHQCHPNTAGENHIGFCRATVFRSHASLEKPKDEQFESDCVILHVKGHAHSHLIQ
QHVEKNSISAEHNNTALNFVHPHNDTRASHESHEHLAKVPVAFVKKELPKDISD
RHTTPVFGCPGLRAVIQPY

>Bcot_FETUA2z

MNSLVALVLLGQIIGSTLSTQVRGDLQCDDKATKEWTDIGVRYINEHKLHGYKY
ALNVIKNIVVTSRDRDLVAVFLKLNLLETECHVLDPTPVKNCTVRTQHNHAVEM
DCDVNIMFNVDTFKEDVFVKCHSTPDSVENVRRNCPKCPILLPSNNPQVVDSVE
YVLNKHNEQLSDHVYEVLEISRGQHKYEPEAYYVEFAIVEVNCTAQEAHDDHH
HCHPNTAGEDHIGFCRATVFRSHATLEKPKDEQFESDCVILHVKKGHAHSHLIQQ
HVEKESISPEHNNTALNLAHPHNDTSTSHESHEHLAKVPVAFVKKELSKDISDRH
TTPVKGCPGKVHHFEL*

>Bcot_FETUA2za
MNSLVALMLLGQIIGSTLSSQVRGDLECNDKDAKEWTDVGVRYINEHKLHGYK
YALNVIKNIVVTSRDRDWVAVFLKLNLLETECHVLDPTPVKNCAVRTQHNHAV
EMDCDVNIMFNVDTFKEDVFVKCHSTPDSVENVRRNCPKCPILLPPNHPQVVDS
VEYVLNKHNEKLSGHVYEVLEISRGQHKYEPEAYYVEFAIVEVNCTAQEAHDDH
HHCHPNTAGEDHIGFCRATVFRSHATLEKPKDEQFESDCVILHVKKGHAHSHLIQ
QHVEKESISPEHNNTALNLAHPHNDTSTSHESHEHLAKVPVAFVKKELSKDISDR
HTTPVKGCPGKVHHFEL*

>Bfon_FETUA1
MNSLVALVLLGQIIGSTLSHHLQSHVDCNGEDAEKWADMAVHYINEHNQHGYK
CALNVINEIRLLPRRPHGTIVFLELKVLETVCHVLDPTPIENCTVRPQHYHAVGGD
CDVKIIHEEGGDKVIGAKCYSDPDSVEDVRRNCPKCPILLSLNDPQVVDSVEYVL
NKHNEKVSGHVYEVLEISRGQHKYEPEAYYVEFAIVEVNCTAQEAHDDHHQCH
PNTAGENHIGFCRGTVFRSHASLEKPKDEQFESDCVIFDVKDGHAHSHLIEHHVG
KYSTSPGHNNTVLNLVHSHNHTSASHESHSHEHVAEVPVAVAKREVPKDIPHDH
THPVKLCPGKVHHFEL*

>Bfon_FETUA2g
MNSLVALVLLGQIIGSTLSSQVRGDLECDEKDAKEWTDVGVRYINEHKLHGYKY
ALNVIKNIVVVPWDGDWVAVFLKLNLLETECHVLDPTPVKNCTVRTQHNHAVE
MDCDVKIMFNVDTFKEDVFAKCHSTPDSVENVRRNCPKCPILLPSNNPQVVDSV
EYVLNKHNEKLSGHVYEVLEISRGQHKYEPEAYYVEFAIVEVNCTAQEAHNDHH
QCHPNTAGENHIAFCRATVFRSHASLEKPKDEQFESDCVILHVKQGHAHSHLIQQ
HVEKDSISPEHNNTALNLIHPHNDTSTSHESHEHVAEVPVAFVKKELPKDISDRHT
TPVKGCPGKVLHFGL*

>Bfon_FETUA2h
MNSLVALVLLGQIIGSTLSSQVREDLECNDETAKWWTDIGVRYINEHKLHGYKY
TLNVIKNIVVTSRDRDWVAVFLKLNLLETECHVLDPTPVKNCAVRTQHNHAVE
MDCDVNIMFNIETYKKDVFVKCHSTPDSVENVRRNCTKCPILLPSNNPQVVDSV
EYVLNKHNEQISGHIYEVLEISRGQHKYEPEAYYVEFAIVEVNCTAQEAHDDHH
QCHPNTAGENHIAFCRATIFRSHASLEKPKDEQVESDCVILHVKKGHAHSHLIQQ
HVEKDSISPEHNNTALNLVHPHNDTSTSHESHEHLAEVPVAFVKKELSKDISDRH
TTPVKGCPGKVLHFQL*

>Bfon_FETUA2i

MNSLVVLVLLGQIIGSTLSSQVRGDLECDEKDAKEWTDVGVRYINEHKLHGYKY
ALNVIKNIVVVPWDGDWVAVFLKLNLLETECHVLDPTPVKNCTVRTQHNHAVE
MDCDVKIMFNVDTFKEDVFAKCHSTPDSVENVRRNCPKCPILLPSNHPQVVDSV
EYVLNKHNEKLSGHVYEVLEISRGQHKYEPEAYYVEFAIVEVNCTAQEAHDDHH
QCHPNTAGEDHIGFCRATVFRSHASLEKPKDEQFESDCVILHVKEGHAHSHLIQQ
HVEKDSISPGHNNTALNLVHPHNYTGTSTSHESHEHLAEVPVAFVKKELPKDISD
RHTTPVKGCPGKVHHFEL*

>Bfon_FETUA2j
MNSLVALVLLGQIIGSTLSTQVRGDLQCDDKATKEWTDIGVRYINEHKLHGYKY
ALNVIKNIVVTSRDRDLVAVFLRLNLLETECHVLDPTPVKNCTVRTQHNHAVEM
DCDVNIMFNVDTFKEDVFVKCHSTPDSVEDVRRNCPKCPILLSLNDPQVVDSVE
YVLNKHNEKLSGHVYEVLEISRGQHKYEPEAYYVEFAIVEVNCTAQEAHDDHH
HCHPNTAGEDHIGFCRATVFRSHASLEKPKDEQFESDCVILHVKKGHAHSHLIQQ
HVEKESISPEHNNTALNLAHPHNDTSTSHESHEHLAKVPVAFVKKELSKDISDRH
TTPVKGCPGKVLHFGL*
